# In Vivo Direct Reprogramming: Current Progress and Future Prospects from Mechanisms to Therapeutic Application

**DOI:** 10.1002/advs.77370

**Published:** 2026-09-21

**Authors:** Rishabh Deo Singh, Mauro Calvoli, Kyeong Kyu Kim

**Affiliations:** ^1^ Department of Precision Medicine Sungkyunkwan University (SKKU) School of Medicine Suwon Republic of Korea; ^2^ Biomedical Institute for Convergence at Sungkyunkwan University (BICS) Suwon Republic of Korea

**Keywords:** functional recovery, gene regulatory network, in vivo reprogramming, multimodal reprogramming, tissue microenvironment

## Abstract

Direct reprogramming, which converts somatic cells from one lineage to another without passing through a pluripotent state, represents a promising therapeutic strategy for regenerative medicine. Recent advancements in identifying reprogramming factors and understanding molecular barriers have enabled efficient generation of therapeutically relevant cells. Yet, the clinical application of in vitro‐derived reprogrammed cells is hampered by the immature phenotype, hostile microenvironments, low engraftment, and poor survival after transplantation. In vivo direct reprogramming of tissue‐resident cells within their native tissue microenvironment offers a compelling alternative to overcome these barriers. Harnessing biochemical and biophysical cues of the tissue microenvironment, this approach facilitates the acquisition of molecular and functional features resembling endogenous cells. This review summarizes the current understanding of in vivo direct reprogramming, covering mechanisms, key reprogramming factors, and delivery strategies, and explores therapeutic applications across organ systems. Finally, key challenges such as delivery efficiency, incomplete understanding of tissue cues, and limited mechanistic insights, along with emerging strategies, are discussed.

## Graphical abstract

This review examines the potential of in vivo direct reprogramming in regenerative medicine for functional tissue restoration, highlighting the role of tissue‐resident cues in generating functionally mature reprogrammed cells from lineage‐related cells. It contains a discussion on mechanisms, reprogramming factors, delivery approaches, and applications across organ systems, along with a discussion on key challenges and future direction.

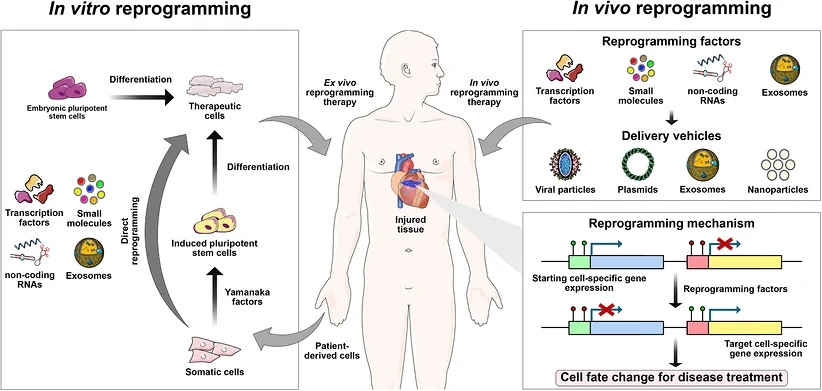

## 1 Introduction

The discovery of cellular plasticity in somatic cells through pioneering studies involving nuclear transfer and cell fusion prompted a reassessment of the concept of cellular differentiation [1, 2, 3, 4]. Subsequently, the direct conversion of mouse embryonic fibroblasts into myoblasts through the transcription factor (TF) MyoD established the foundation of direct reprogramming, enabling the generation of therapeutically relevant cell types without relying on embryonic stem cells [5]. The field of cell reprogramming advanced further when Takahashi and Yamanaka identified a combination of four transcription factors (Oct4, Sox2, Klf4, and c‐Myc) capable of reprogramming somatic cells into induced pluripotent stem cells (iPSCs) [6]. iPSCs possess the potential to be differentiated into various cell types, offering a transformative platform to restore the functionalities of damaged organs without necessitating whole‐organ transplantation [7, 8, 9, 10]. Based on iPSCs‐derived cells, several cell‐based therapies have been developed, received regulatory approval, and are undergoing clinical trials [9, 11, 12]. Despite the success of preclinical and phase I clinical trials, the widespread adoption of iPSC‐derived cells in a clinical setting faces challenges such as tumorigenesis risk, reprogramming and differentiation kinetics, and survival and functional integration of the transplanted cells [13, 14, 15].

Direct reprogramming or trans‐differentiation, which converts one somatic cell type into another, has emerged as an alternative approach to iPSC‐based therapy [2, 3, 5, 6, 16, 17, 18, 19] (Figure 1A). Owing to its rapidity, efficiency, and relative safety, direct reprogramming has gained substantial attention and spurred the development of numerous reprogramming strategies [20, 21, 22]. Although directly reprogrammed cells avoid multiple bottlenecks associated with iPSC‐based cellular therapeutics, the clinical outcome of these in vitro reprogrammed cells is limited due to impaired functionality resulting from incomplete or immature reprogramming under in vitro conditions [22, 23, 24]. Moreover, low tissue homing and integration, and poor long‐term survival post‐transplantation represent additional limitations [25, 26, 27, 28, 29]. Consequently, developing an efficient reprogramming strategy is crucial for generating mature cells and improving tissue integration after transplantation.

**FIGURE 1:**
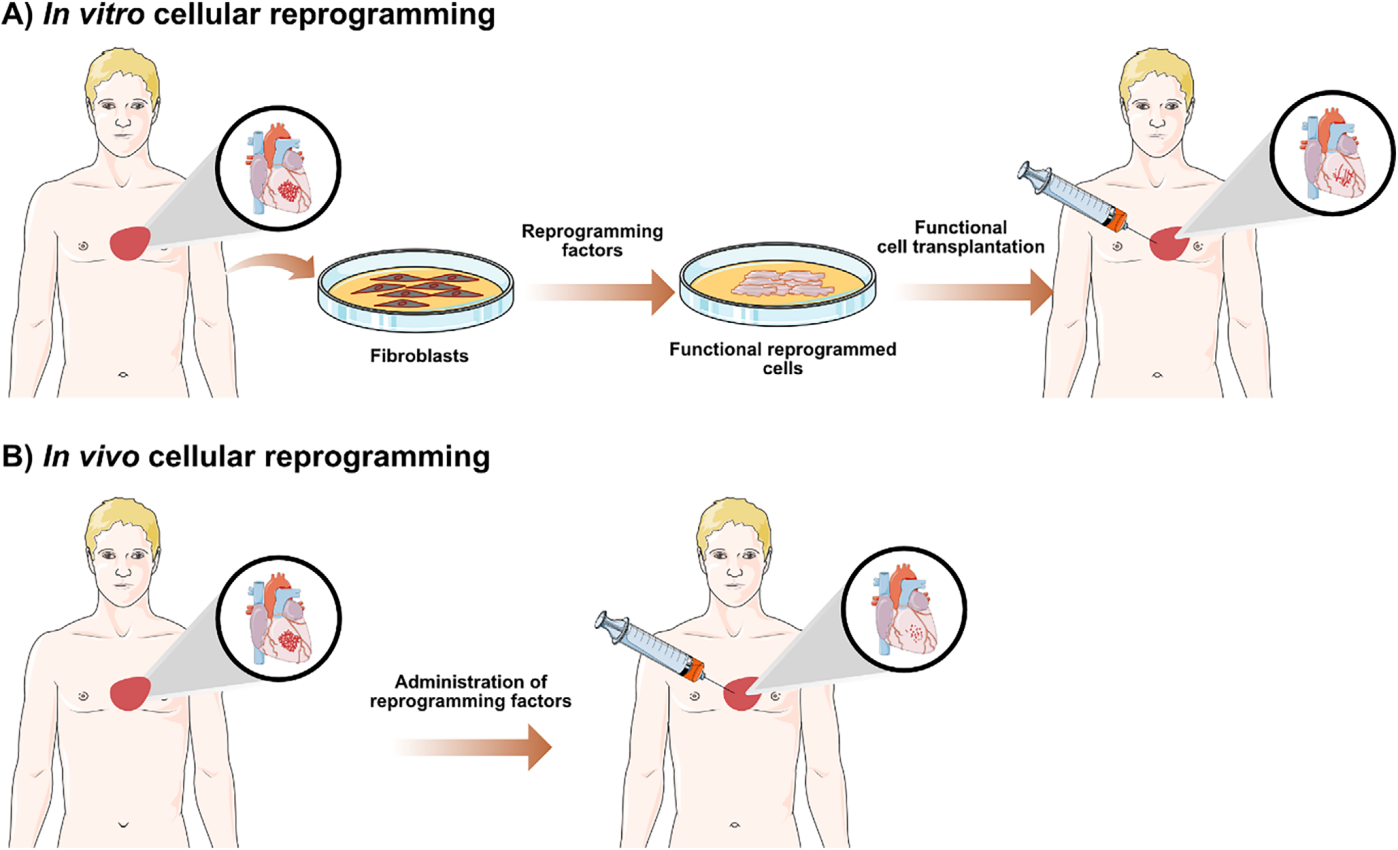
Overview of direct reprogramming and its application in a clinical setting. (A) Schematic representing the key steps of direct cellular in vitro reprogramming: Isolation of patient‐specific cells, their reprogramming to functional target cell types, and subsequent transplantation at the injury site for the functional recovery of damaged organs. (B) Schematic showing the direct administration of reprogramming factors at the site of injury to promote in vivo direct reprogramming and facilitate the integration of reprogrammed cells, thereby promoting functional recovery of damaged organs. Figures were drawn using pictures from Servier Medical Art, by Servier, licensed under a Creative Commons Attribution 4.0 Unported License. Figures were designed or edited using Affinity Designer, a vector graphics software, to create high‐resolution vector illustrations and infographics.

To overcome the limitations of current cell transplantation therapies, in vivo direct reprogramming of tissue‐resident cells capable of replacing damaged cells has emerged as a promising approach. (Figure 1B). In vivo direct reprogramming utilizes the intrinsic molecular similarities between lineage‐related cells to reset molecular signatures and produce mature cells compared to their in vitro counterparts [30, 31]. This outcome can be attributed to the presence of pro‐developmental [32, 33] and regenerative cues within the tissue microenvironment, which facilitates the induction of mature cells [22, 34, 35]. Moreover, in vivo direct reprogramming has been shown to enhance cell‐tissue integration, thereby promoting functional recovery following tissue abnormalities (Figure 2) [30, 36, 37].

**FIGURE 2:**
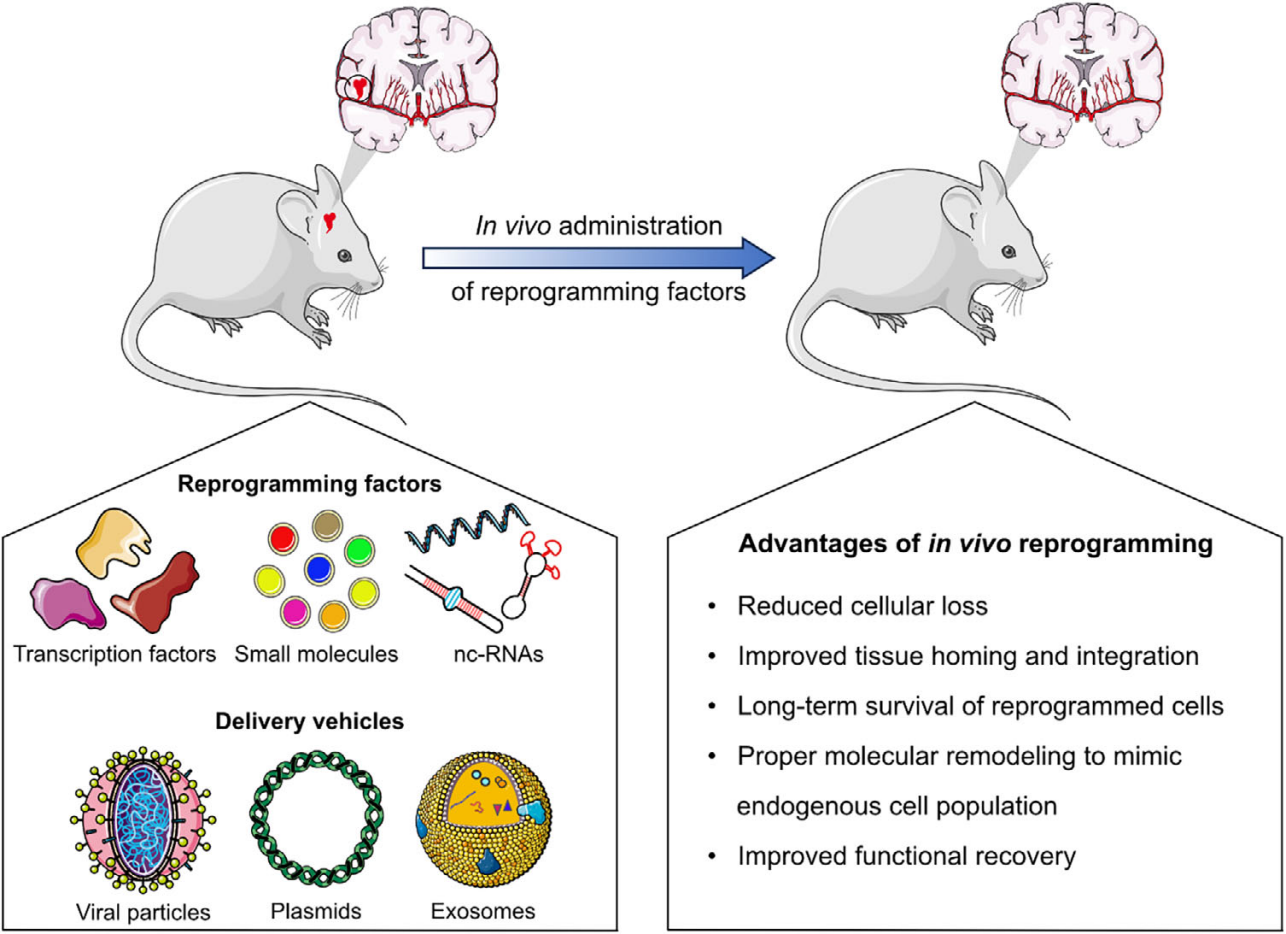
Reprogramming factors, delivery vehicles, and advantages of in vivo reprogramming. In vivo reprogramming is facilitated by various reprogramming factors, such as transcription factors, small molecules, and non‐coding RNAs (nc‐RNAs). Various delivery vehicles, such as viruses, plasmids, and exosomes used either in their native or engineered form to deliver the factors to the target organ in vivo. The successful in vivo reprogramming mediated by the reprogramming factors has several advantages over in vitro direct reprogramming, such as improved tissue integration, generation of mature cells, and improved functional recovery. Figures were drawn using pictures from Servier Medical Art, by Servier, licensed under a Creative Commons Attribution 4.0 Unported License. Figures were designed or edited using Affinity Designer, a vector graphics software, to create high‐resolution vector illustrations and infographics.

Accordingly, in vivo direct reprogramming has seen rapid adoption and development and is emerging as a key field in regenerative medicine. However, despite its potential, there are still relatively few comprehensive reviews addressing this topic, underscoring the need for a timely synthesis of current knowledge. This review aims to fill that gap by examining the various strategies and reprogramming factors employed for direct in vivo reprogramming, as well as discussing the challenges that limit its clinical translation. In addition, it explores future perspectives, highlighting the development of novel reprogramming strategies and ongoing scientific advancements that may accelerate progress in this field.

## 2 The Overall Mechanism of Direct Reprogramming

Direct reprogramming involves a comprehensive reset of epigenetic and transcriptomic profiles, which are regulated by a gene regulatory network (GRN) established during development [38, 39]. A GRN, comprising lineage‐specific TFs, signaling pathways, non‐coding RNAs, and chromatin remodelers, is hierarchically activated during development and confers cells with stable and unique molecular signatures (Box 1) [40, 41, 42]. At the center of the GRN is the self‐sustaining core regulatory circuit, which is activated by the lineage‐specific master regulators and is responsible for establishing cell identity [43, 44]. During direct reprogramming, reprogramming factors activate lineage‐specific master regulators and signal transduction machinery to form the core regulatory circuit and drive the induction of downstream components to establish a complete lineage‐specific GRN [21, 45]. The core regulatory circuits are often governed by a class of TFs known as pioneering factors that can interact with heterochromatin regions to induce local chromatin opening [21, 46]. For example, during TF‐mediated reprogramming of fibroblasts to neurons, Ascl1 works as a pioneer factor that binds to neural‐lineage‐specific targets independent of chromatin accessibility [47]. The binding of Ascl1 leads to the recruitment of master regulators Brn2 and Mytl1, which form the core GRN along with Ascl1 to induce a neuron‐specific GRN and generate reprogrammed neurons. The pioneering activity of these factors begins with chromatin scanning, mediated by non‐specific electrostatic interactions with the phosphodiester backbone, to identify specific DNA‐binding sites [48, 49]. Initial binding results in local chromatin decompaction, leading to DNA distortion and unwrapping, thereby increasing accessibility to other proteins [50, 51, 52, 53]. The site‐specific interaction of pioneer factors is followed by the recruitment of transcriptional co‐regulators, which facilitates chromatin remodeling and induces a transcriptionally permissive state [54]. These co‐regulators, classified as either co‐activators or co‐repressors, influence chromatin packaging through chemical modification of histones or ATP‐dependent chromatin remodeling [54, 55]. The epigenetic modeling is accompanied by the induction and binding of other lineage‐specific master regulators, which marks the induction of a new lineage‐specific GRN [56]. During this process, the master regulators also suppress the expression of non‐lineage‐specific TFs, either by redirecting the transcriptional machinery away from the transcriptionally active site or by modulating the epigenetic state [57, 58, 59].

**Box 1: Gene regulatory network**

A gene regulatory network (GRN) is composed of complex interactions among chromatin, transcription factors, genes, non‐coding RNAs, metabolites, and other regulatory elements that collectively define cellular identity. These components are arranged in a hierarchical structure, where molecular programs at each level correspond to distinct stages of development. During early development, lineage‐specific pioneer factors, transcription factors capable of binding heterochromatin, initiate a self‐regulatory positive feedback loop that establishes the core of the GRN. Activation of this loop triggers the subsequent induction of downstream GRN components, guiding the establishment of cellular identity. The precise installation of each molecular program is essential for the proper initiation of the next stage in the hierarchy, with any disruption leading to an aberrant cell identity or functionality. As progression through the hierarchy continues, cellular plasticity decreases, restricting the potential for identity changes and specifying the cells to a particular subtype. Intermediate molecular programs within the GRN are responsible for specifying subtypes, which are activated after the broader cell identity has been set. The GRN ultimately concludes with peripheral networks or terminal molecular programs that determine the subtype and functionality of the cells. Understanding this hierarchical organization provides a framework for identifying and targeting specific molecular programs to achieve direct reprogramming, enabling the generation of progenitor, immature, or fully specialized cell types.

The initial binding and epigenetic modifications induced by pioneer factors lead to the induction of downstream lineage‐specific master regulators that activate other GRN components. A successful reprogramming trajectory culminates in a peripheral GRN, which governs the terminal fate of reprogramming and is responsible for the maturity and functionality of reprogrammed cells [21]. The upstream regulators in the hierarchy induce the expression of GRN terminal genes to regulate the maturation of reprogrammed cells [60]. For instance, during pancreatic beta cell reprogramming, Mafa and Errγ are often considered to constitute peripheral GRN that regulate insulin release following glucose sensitization [61, 62]. In response to glucose stimulation, Mafa binds to and induces the expression of insulin and effectors of glucose‐sensitive secretion, whereas Errγ induces the expression of genes that regulate mitochondrial oxidative metabolism to grant the reprogrammed cells maturity [60]. In summary, direct reprogramming is orchestrated by the core regulatory circuit activated by either transcription factors, small molecules or non‐coding RNAs, that overcome epigenetic barriers, allowing master transcription factors to establish lineage‐specific GRNs and drive the maturation and functionality of reprogrammed cells.

## 3 In Vitro‐Based Reprogramming Factors and Their Working Mechanism

An increasing understanding of cell‐specific GRNs and their regulatory capabilities has facilitated the identification of numerous factors relevant to direct reprogramming (Table 1). Many of the pioneering in vitro studies have served as the basis for establishing reprogramming strategies and understanding the mode of action of several reprogramming factors. This section discusses various categories of reprogramming factors, including TFs, small molecules, non‐coding RNAs, and exosomes, and elucidates the roles and mechanisms underlying direct reprogramming based on in vitro studies. In addition, advantages and limitations of each reprogramming factor involved in direct reprogramming are compared in Table 1.

**TABLE 1:** Comparison of reprogramming factors.

Sl. No	Reprogramming factor	Examples of reprogramming factors	Mechanism of action	Range of in vivo efficiencies	Advantages	Limitations
1	Transcription factors [21]	Ascl1, LMX1a, and Nurr1 (Glial cells to neurons) [210] Gata4, Mef2c, and Tbx5 (Cardiac fibroblasts to cardiomyocytes) [65]	Binding to regulatory regions of target genes to induce lineage‐specific GRNs	Cardiac fibroblasts to cardiomyocytes: ∼5% [65] Exocrine to Beta cells: ∼20% [143] Astrocytes to neurons: ∼7% [153]	Known binding sites and better understanding of downstream effects Targeting different levels of hierarchy in the GRN controlling developmental stage Availability of several delivery approaches	Risk associated with genomic integration and DNA damage Unknown functionalities from sustained expression in reprogrammed cells Challenges in dose optimization inside target cells
2	Small molecules [20]	CHIR99021, RepSox, Forskolin, VPA, Parnate, TTNPB, and Rolipram (Cardiac fibroblasts to cardiomyocytes) [207] SB431542 and NKH477 (White adipocytes to brown adipocytes) [209]	Activation of signaling pathways and epigenetic modifications inducing lineage‐specific GRNs	Cardiac fibroblasts to cardiomyocytes: ∼1% [207] Astrocytes to neurons: ∼11% [208]	Transient effect with lower risk of continuous exposure Controlled dose and exposure time of reprogramming factors Minimal risk of insertional mutagenesis and DNA damage Controlled targeting of different GRN components	Off‐target effects hindering pathway synergy and downstream effects Lack of a precise and targeted delivery approach Need for better understanding of PK/PD of multiple drugs delivered simultaneously Short half‐life requiring repeated administration
3	Non‐coding RNAs [87]	miRNAs 1, 133, 208, and 499 (Cardiac fibroblasts to cardiomyocytes) [106] shRNA against PTB protein (Astrocytes to neurons) [119]	Degradation and translational repression of target mRNAs to induce cell fate change	Cardiac fibroblasts to cardiomyocytes (miRNA): ∼1% [106] Astrocytes to neurons (shRNA): ∼20% [119]	Transient effect with reduced risk of continued exposure Non‐integrative with minimal risk of insertional mutagenesis Availability of several delivery approaches Modifiable for increased target specificity	Low stability and short half‐life in vivo Unknown targets limiting mechanistic understanding of reprogramming Transient expression requiring repeated administration
4	Exosomes [185]	ADSCs‐derived exosomes (M1 to M2 macrophage) [225] Exosomes carrying CRISPR‐Cas9‐based transcriptional activator for Hnf4α (Hepatic stellate cells to hepatocytes) [201]	Delivery of coding/non‐coding RNAs and proteins to rewire molecular signatures and induce cell fate change	Tumor‐associated macrophages (TAMs) to M1 macrophages (exosomes carrying ASO‐STAT6): ∼50% [376] TAMs to M1 macrophages (M1‐derived exosomes): 5∼10% [377]	Biocompatible and less immunogenic Ability to cross biological barriers Engineered loading of biologics as cargo Minimal risk of insertional mutagenesis Surface modifiable for precise tissue/organ targeting	Heterogeneity and inconsistency in the cargo Limited understanding of biologics in cargo during reprogramming Short half‐life requiring repeated administration

### 3.1 Transcription Factors for Reprogramming

TF‐based direct reprogramming uses pioneer factors that are known to regulate lineage specification during embryonic development. These factors bind to DNA in a sequence‐specific manner, initiating the activation of key transcriptional regulators and establishing the core GRN essential for cell fate change. In the presence of pioneer factors, derepression of lineage‐specific master regulators signals the onset of a new GRN that governs temporal gene expression and dictates the acquisition of target cell identity. The developmental stage at which pioneer factors act, along with the specific GRNs they initiate, influences the reprogramming trajectory and determines the characteristics of intermediate cell states generated during direct reprogramming [21, 63, 64].

In one of the earliest studies, Ieda et al. [65] used a combination of cardiac‐specific TFs, Gata4, Mef2c, and Tbx5 (GMT) to directly reprogram fibroblasts into mature and functional cardiomyocytes in vitro. Through systematic screening of 14 cardiac developmental regulators, a specific combination of GMT was identified, which effectively reprogrammed postnatal cardiac and dermal fibroblasts into functional cardiomyocytes. To initiate the cell state transition from fibroblasts to cardiomyocytes, Mef2c and Tbx5 engage and remodel the heterochromatin, making the landscape accessible to cardiac‐specific TFs, including Gata4, consequently leading to the induction of cardiac‐specific genes. For direct reprogramming, TFs are known to influence each other's binding patterns to streamline the reprogramming trajectory and induce the desired GRN. For example, Gata4 is involved in lineage specification of both mesodermal and endodermal lineage cells during development [66]. Similarly, Tbx5 and Mef2c are involved in regulating limb‐ and neural‐lineage specifications, respectively [67, 68]. However, when used in combination during the reprogramming process, these TFs refine each other's binding patterns to facilitate the induction of cardiac‐specific genes. Molecular analysis of fibroblasts undergoing in vitro direct cardiac reprogramming revealed distinct binding patterns of these reprogramming factors when transfected alone compared to when used in combination [69]. Apart from influencing the binding patterns, the synergistic effects also ensure the suppression of other lineages. For instance, during in vitro direct neuronal reprogramming, ectopic expression of Ascl1 alone leads to extensive chromatin remodeling and initiation of a neural program [56]. However, cells expressing Ascl1 alone failed to fully convert into mature neurons and instead adopted a predominantly myogenic transcriptional profile. The introduction of two other neural master regulators, Brn2 and Myt1l, suppresses the myogenic program and generates functionally mature neurons by inducing neuron‐specific GRN.

### 3.2 Small Molecules for Reprogramming

Compared to TF‐based approaches, small‐molecule‐based approaches offer several advantages, including better reprogramming kinetics and efficiency, as well as precise control over reprogramming dynamics through adjustable concentration and exposure duration [70, 71]. These attributes make small molecules particularly well‐suited for dissecting reprogramming intermediates and elucidating the molecular mechanisms that govern the reprogramming process. By modulating signaling pathways and the epigenetic landscape, small molecules can reshape the cell transcriptome, inducing the expression of several transcription factors and non‐coding RNAs that can influence the target GRN [72, 73, 74]. Moreover, several signaling pathway‐associated effector molecules and TFs act synergistically and co‐localize with lineage‐specific master regulators, thereby influencing cell fate decisions [75, 76, 77]. In addition to pathway activation, small‐molecule inhibitors also facilitate direct reprogramming by suppressing TFs associated with alternative lineage programs or biological processes that serve as molecular barriers to reprogramming [78, 79].

Small‐molecule cocktails for direct reprogramming can be rationally designed to mimic the developmental signaling cues of the target cell type, thereby coordinating the activation of transcriptional effectors and lineage‐specific master regulators. Yin et al. [80] used a chemical cocktail containing CHIR99021 (Wnt activator), SB431542 (TGFβ inhibitor), DAPT (Notch inhibitor), and LDN193189 (BMP inhibitor) for in vitro direct reprogramming of human fetal astrocytes from cortical tissue to cortical neurons. Several studies have demonstrated the role of the strength and duration of signaling pathways like Nodal, Wnt, and bone morphogenetic protein (BMP) signaling in neural lineage specification [81, 82, 83]. During the reprogramming process, the molecules acted synergistically to downregulate glial genes and activate neural lineage‐specific master regulators, which are known to regulate the development of cortical neurons [84]. Interestingly, many of the developmental regulators like Emx2 and Cux2 have been reported to be the direct or downstream targets of the signaling pathways targeted by the small molecules [84, 85, 86]. The activation of these developmental regulators led to the suppression of the glial GRN and activation of the neural lineage GRN to generate reprogrammed neurons.

### 3.3 Non‐Coding RNAs for Reprogramming

Out of the 90% of the eukaryotic genome that is transcribed, only 1–2% of the transcripts encode proteins. The remaining transcripts are non‐coding RNAs (ncRNAs) that do not translate into proteins, but a bulk of that portion is known to regulate gene expression [87, 88, 89]. These ncRNAs are closely associated with development and tissue homeostasis, and dysregulation of their expression is associated with abnormalities in complex cellular processes [90, 91, 92]. The mechanistic roles of ncRNAs in regulating gene expression are related to their ability to regulate transcription, modify chromatin, and induce post‐transcriptional modifications [89, 93, 94, 95, 96]. Owing to their roles in epigenetic, transcriptional, and translational machinery, various ncRNAs have been exploited to activate specific GRNs for direct reprogramming.

#### 3.3.1 MicroRNAs

MicroRNAs (miRNAs) are small non‐coding RNAs, approximately 22 nucleotides long, that play a crucial role in the post‐transcriptional regulation of gene expression. They form an RNA‐induced silencing complex (RISC) with the Argonaute family of proteins, which repress translation by interfering with 7‐methylguanine (7mG) cap recognition and ribosomal subunit interactions. The miRISC complex can also induce messenger RNA (mRNA) decay through polyA deadenylation, 7mG decapping, and mRNA cleavage [97]. Recent studies have revealed that miRNAs can also enhance translation by binding to the 3′ or 5′ untranslated regions (UTRs) of mRNAs and recruiting the translational machinery [98, 99, 100]. Additionally, miRNAs contribute to epigenetic modulation by targeting enzymes responsible for numerous active and repressive chromatin marks [101, 102, 103].

miRNAs are essential for embryonic development and differentiation [104, 105], making them valuable tools for cell fate reprogramming in regenerative medicine. Jayawardena et al. [106] demonstrated this potential by using a combination of four miRNAs (1, 133, 208, and 499) (miR combo) to generate functional cardiomyocytes from cardiac fibroblasts in vitro. These miRNAs were selected based on their known roles in cardiac lineage development, differentiation, and function [107, 108]. During development, miR‐1 and miR‐133 have been reported to induce mesodermal lineage and myogenesis while simultaneously suppressing genes of other lineages [109, 110]. Within the context of direct cardiac reprogramming, miR‐133 further enhances lineage conversion by inhibiting fibroblast‐specific molecular signatures [111]. Meanwhile, miR‐208 and miR‐499 support the acquisition of cardiomyocyte identity by regulating myosin expression and contributing to muscle contractile function [112], thus reinforcing the overall reprogramming process.

#### 3.3.2 Short Hairpin RNAs

Short hairpin RNAs (shRNAs) are non‐coding functional RNAs that utilize the miRNA‐processing machinery [113, 114]. These double‐stranded (ds) RNAs form a hairpin structure and are recognized by RNA interference (RNAi) machinery for further processing [115]. In the cytoplasm, the RNase III enzyme Dicer processes shRNAs into active small interfering RNAs (siRNAs) of 20–25 nucleotides [116]. The resulting siRNAs form a complex with RISC, guiding the targeting of the complementary mRNA and inducing cleavage in the center of the duplex region at a site ten nucleotides away from the 5′‐end of the siRNA [117]. Although shRNAs and miRNAs share similar physicochemical properties and are processed via the RNAi pathway, shRNAs typically target a single mRNA because of their sequence complementarity with the target mRNA. In contrast, miRNAs generally regulate multiple targets through partial complementarity [118].

Qian et al. [119] reported the potential of shRNAs in direct reprogramming by converting mouse astrocytes into neurons both in vitro and in vivo. This direct reprogramming was achieved by repressing the expression of the RNA‐binding protein Ptb (also known as Ptbp1), which plays a pivotal role in embryonic development, cell cycle progression, transcriptional regulation, and neural fate determination. The repression of Ptb by shRNA activates neuron‐specific transcriptional programs, thereby promoting neural induction and maturation [120].

#### 3.3.3 Antisense Oligonucleotide

Antisense oligonucleotides (ASOs) are chemically synthesized, single‐stranded, artificial polymers, typically 18–30 nucleotides long, designed to silence genes by targeting specific RNAs via base complementarity [121, 122]. When bound to their targets, ASOs facilitate the recruitment of RNase H or Argonaute 2, leading to target cleavage. Alternatively, ASOs can inhibit the assembly of translational complexes and induce alternative splicing without degrading the target [123, 124].

Qian et al. [119] used Ptb ASO to transiently suppress Ptb, enabling the in vivo conversion of mouse astrocytes into functional neurons. This conversion was attributed to the self‐reinforcing nature of the PTB‐regulatory loop. Their study showed that stereotactically injected PTB ASO‐induced dopaminergic neurons with neurophysiological properties alleviated the 6‐OHDA lesion‐induced phenotype three months after injection [119].

### 3.4 Exosomes

Released by most of the cells, exosomes are fundamental components of the secretome and play an important role in cell–cell communication and other physiological processes by exchanging biologically active components between cells [125, 126]. Exosomal cargo includes lipids, non‐coding RNAs, proteins that can function as TFs, and ligands for signaling receptors, capable of inducing changes in molecular signatures of recipient cells [127, 128]. In addition to altering the transcriptional landscape or protein composition, exosomes can modulate the epigenetic state of cells by releasing enzymes or non‐coding RNAs capable of modifying epigenetic marks [129, 130, 131]. In the context of direct reprogramming, exosome‐mediated delivery of appropriate bioactive components can potentially target and induce the desired GRN to induce a reprogramming effect [132].

Mandal et al. [133] used insulinoma‐derived exosomes to convert exocrine cells into pancreatic beta‐like cells in vitro. Sequencing analysis of the miRNA content in exosomes and their parent cells revealed the enrichment of miRNAs involved in pancreatic development. Out of multiple miRNAs enriched in the exosomes, a combination of miR‐127 and miR‐709 was found to be sufficient to directly reprogram exocrine cells to pancreatic beta cells. This enrichment is potentially due to selective packaging and sorting mechanisms that regulate miRNA inclusion in exosomes [134].

## 4 Delivery Methods

The efficacy of reprogramming factors in inducing in vivo reprogramming depends on their precise delivery into target cells, as well as the appropriate dose and retention time within these cells. Delivery of reprogramming factors can be achieved either by direct administration into the bloodstream or tissues, relying on passive diffusion, or by employing delivery vehicles that enhance stability, targeting, and cellular uptake. Different delivery vehicles have been developed for in vivo reprogramming, considering their ease of administration, cellular uptake, release of reprogramming factors within host cells, and effects on host cell genome integrity. In this section, we describe various delivery vehicles, including viruses, plasmids, nanoparticles, and exosomes, which are routinely used to deliver reprogramming factors in vivo. In addition, their advantages and limitations in delivering the reprogramming factors are compared in Table 2.

**TABLE 2:** Comparison of delivery methods.

Sl. No	Delivery Vehicle	Payload capacity	Reprogramming efficiency of different delivery vehicles	Detectable presence of reprogramming factors	Advantages	Limitations
1	Adenoviral vector [136, 137]	High (transgene cassettes up to 36 kb in length)	Exocrine to beta cells (transcription factors): ∼20% [143] Hepatic cells to beta cells (transcription factors): 6∼10% [216]	Short (Weeks to months)	High transduction efficiency for both dividing and non‐dividing cells Episomal persistence Broad tissue tropism	Pre‐existing immune response Clearance of virus‐transduced cells Cellular toxicity
2	Adeno‐associated viral vector [136, 144]	Low (transgene cassettes up to 4.6 kb in length)	Glia cells to neurons (transcription factors): ∼20% [210] Astrocytes to neurons (shRNA):∼20% [119]	Long (Months to years)	Single‐dose administration Broad tissue tropism Low immunogenicity	In vitro models do not reflect in vivo efficacy Genomic integration Cellular toxicity Long‐term safety concerns
3	Lentiviral vector [136, 146]	Low to moderate (transgene cassettes up to 9 kb in length)	Cardiac fibroblasts to cardiomyocytes (miRNA): ∼12% [378] Astrocytes to neurons (transcription factors): 6∼8% [153]	Long (Months to years)	Incorporation of multiple genes in a single vector High transduction efficiency for postmitotic and quiescent cells Low immunogenicity	Risk of insertional mutagenesis Induces aberrant splicing events Ability to activate neighboring genes Genomic integration
4	Plasmid [154, 161, 162]	High (transgene cassettes up to 100 kb in length)	Callosal neurons to corticofugal neurons (transcription factor): ∼40% [164] Hepatocytes to insulin‐positive cells (transcription factors): 10∼20 cells [379]	Short (Days to weeks)	Low cost, easy manipulation, and long shelf‐life Incorporation of multiple genes within a single vector Low immunogenicity Minimal risk of insertional mutagenesis	Lower efficiency compared to viral vectors Transient transgene expression diluted by cell division Risk of transferring antibiotic resistance genes to human microbiota
5	Nanoparticle [168, 173, 175, 177]	Low to moderate (∼20,000 DNA strands/1–3 mRNA molecules per particle)	Cardiac fibroblasts to cardiomyocytes (mRNA):∼1.5% [181] Cardiac fibroblasts to cardiomyocytes (transcription factors): ∼25% [380]	Short (Days to weeks)	Ability to deliver multiple cargoes Surface modifiable for tissue‐specific targeting Low immunogenicity Minimal risk to genomic integrity	Transient expression requiring multiple administrations Clearance by the mononuclear phagocyte system Potential off‐target biodistribution
6	Exosome [187, 188, 190, 193, 194]	Low (DNA molecules up to 700 bp in length, ∼1500‐3000 copies of ncRNAs/exosome)	Tumor associated macrophages (TAMs) to M1 macrophages: ASO‐STAT6: ∼50% [376] TAMs to M1 macrophages (M1‐derived exosomes): 5∼10% [377]	Transient (Short half‐life in the circulation, 90% cleared in 5 mins. Cargo half‐life ranges from hours to days)	Biocompatible with low immunogenicity Ability to deliver multiple cargo Engineered for enhanced specificity Ability to cross biological barriers Minimal risk to genomic integrity	Heterogeneity and cargo inconsistency Low circulation half‐life Cargo loading hurdles (efficiency and membrane integrity issues)
7	Direct administration [80, 206, 207]	Moderate to high (4‐7 molecules)	Cardiac fibroblasts to cardiomyocytes (small molecules): 0.6∼1.1% [207] Astrocytes to neurons (Small molecules) ∼10% [208]	Transient (Hours to 1–2 days)	Non‐invasive Convenient for repeated administration Simultaneous delivery of multiple drugs Minimal risk to genomic integrity	Non‐specific effects with potential off‐target activity Repeated administration for continued activity Rapid clearance from the body

### 4.1 Viral Vectors‐Based Delivery

Viral vectors have been extensively used to insert new genetic information into mammalian cells [135]. The viral genome contains genetic information that encodes the proteins responsible for viral replication and structural proteins. The transgene of interest is incorporated into the viral genome by replacing certain portions of the genome during viral production. For the in vivo delivery of reprogramming factors, adenoviruses, adeno‐associated viruses, and lentiviruses are the most commonly used viral vectors. The choice of these viral vectors for in vivo delivery of reprogramming factors depends on their loading capacity, transduction ability, and duration of transgene expression [136].

#### 4.1.1 Adenoviral Vectors

Adenoviruses (Ads) are double‐stranded viruses with a large cargo capacity of up to 36 kb that are widely used for the transient expression of the transgene (Table 2) [136, 137, 138]. These viruses are promising candidates for reprogramming factor delivery because of their ability to efficiently transduce both dividing and non‐dividing cells, target multiple tissues, and facilitate mass production [139, 140]. Once inside the cell, the viral genome enters the nucleus through the nuclear envelope pore complex, where it persists as an episome or extrachromosomal DNA without amplification, allowing for the short‐term expression of the reprogramming factors [141, 142]. Using an adenoviral vector, Zhou et al. [143] delivered a combination of three TFs, Ngn3, Pdx1, and Mafa, to convert pancreatic exocrine cells into beta cells. Purified viral particles were directly injected into the splenic lobes of the dorsal pancreas of the mice. The reprogramming factors transformed >20% of the infected cells into insulin‐positive cells within 10 days of viral delivery, with the effect persisting for >3 months.

#### 4.1.2 Adeno‐Associated Viral Vectors

Adeno‐associated viruses (AAVs) are small single‐stranded DNA viruses with a loading capacity of up to 4.6 kb, often used for sustained expression of transgenes (Table 2) [144]. After entry into the host cell's nucleus, the synthesis of the second strand commences to form a ds genome. This dsDNA persists in the nucleus in an episomal state and can be maintained during cell division because of its close association with the host nuclear DNA [136, 145]. Their persistence inside the host nucleus enables the sustained expression of transgenes, making them a promising vector for the in vivo delivery of reprogramming vectors. Yao et al. [36] used AAV vectors to deliver β‐catenin and the TFs Otx2, Crx, and Nrl to Müller glia (MG) cells, reprogramming MGs into rod photoreceptors in mature mouse retinas. AAVs were administered via intravitreal injection using a microsyringe. Introduction of β‐catenin stimulates MG proliferation, whereas the expression of TFs in proliferating MGs generates rod photoreceptors.

#### 4.1.3 Lentivirus

Lentiviruses are single‐stranded RNA viruses with a loading capacity of up to 9 kb, widely used for long‐term transgene expression through host genome integration (Table 2) [136]. In the cytoplasm of the host cells, viral reverse transcriptase converts single‐stranded RNA into dsDNA, which is then transported to the nucleus, where the viral integrase facilitates genomic integration [146, 147, 148]. Lentiviral vectors possess the ability to express multiple genes from a single vector and transduce both post‐mitotic and quiescent cells, which makes them a plausible candidate for the delivery of reprogramming factors [149, 150, 151, 152]. Su et al. [153] used a lentiviral vector to ectopically express Sox2 in injured spinal cord‐resident astrocytes to reprogram them into neuroblasts. Purified viral particles were manually injected into the spinal cord parenchyma using a Hamilton syringe. Newly generated neuroblasts differentiated into mature neurons and formed neural synapses with pre‐existing motor neurons.

### 4.2 Plasmid‐Based Delivery

Plasmids are circular dsDNA non‐viral vectors with a loading capacity of up to 100kb, often employed for transient or long‐term expression of the transgene based on their design (Table 2) [154, 155, 156, 157, 158]. Following entering the cytosol, plasmids enter the nuclei of dividing cells through the nuclear envelope after they break down during mitosis [159]. Within the nucleus, the plasmid interacts with histone proteins during mitosis and chromatinizes during nuclear envelope formation [159]. In dividing cells, sustained transgene expression requires a mammalian origin of replication to prevent plasmid dilution during cell division, in the absence of which the transgene is transiently expressed [160, 161, 162]. In non‐dividing cells, nuclear import is facilitated by nuclear pore complexes, which can retain an extrachromosomal component in non‐dividing cells, provided that it is stable and protected from a hostile cellular environment [163]. Owing to their ease of design to regulate transgene expression, incorporate multiple genes in a single vector, and large loading capacity, plasmids offer themselves as a compelling platform for the in vivo delivery of reprogramming factors. Rouaux et al. [164] used a plasmid vector for the conversion of post‐mitotic terminally differentiated callosal projection neurons (CPNs) into corticofugal projection neurons (CFPNs). A vector carrying the master regulator of CFPNs, Fezf2, under the *Cdk5r* gene promoter (restricted to migratory post‐mitotic neurons) was electroporated in utero during the early and late stages of post‐mitotic life [165, 166]. Transient Fezf2 expression induces a shift in the molecular identity of CPNs toward CFPNs and a change in axonal connections to corticofugal projections.

### 4.3 Nanoparticles

A nanoparticle (NP) is an organic, inorganic, or hybrid material with dimensions in the nanometer range. NP‐based delivery vehicles have been shown to overcome the limitations associated with conventional drug formulations, ranging from large‐scale problems such as biodistribution to small‐scale problems such as intracellular trafficking [167]. Nanoparticles have shown promising potential for enhancing the efficacy of drugs by improving their stability and solubility, facilitating their transport across cellular membranes, and prolonging their systemic circulation time [168]. The need to encapsulate various types of drugs within these carriers for controlled and targeted release has driven the development of nanoparticles with complex architectures, site‐specific responsiveness, and specialized targeting agents [169, 170, 171, 172]. Consequently, nanoparticles encapsulating various types of drugs, including small molecules, nucleic acids, and proteins, have shown promising results in in vitro and preclinical studies, highlighting their potential to be used for the in vivo delivery of reprogramming factors (Table 2) [173, 174].

Among many NPs, lipid nanoparticles (LNPs) have been extensively investigated for the in vivo delivery of nucleic acid‐based drugs, and multiple formulations have reached clinical settings [175, 176]. The successful delivery of nucleic acids by LNPs to their targets in vivo is determined by the protection of nucleic acids from nucleases, the ability of LNPs formulations to evade the mononuclear phagocytic system, their internalization into target cells, and the release of nucleic acids from endosomes into the cytoplasm [177, 178]. The rational design of LNPs for the efficient delivery of drugs in vivo includes the choice of lipids with cationic or ionizable heads, lipids functionalized with cholesterol or polyethylene glycol (PEG), lipids containing phospholipids, etc [175, 179]. Once they reach the target cells, drug‐loaded nanoparticles are internalized via endocytic pathways, including phagocytosis, clathrin‐, caveolae‐dependent/independent endocytosis, and micropinocytosis [180]. There are also reports of entry via other mechanisms, such as passive diffusion, hole formation, and electroporation [180].

Wang et al. [181] used nanoparticles to specifically target cardiac fibroblasts for direct in vivo reprogramming and cardiac regeneration after injury. Mesoporous silicon nanoparticles loaded with miRNAs were coated with fibrinogen‐derived homing (FH) peptide‐modified neutrophil‐mimicking membranes for the precise targeting of the injured tissue. A nanoparticle conjugate carrying miRNAs with reprogramming abilities was intravenously injected into mice with myocardial infarction. Neutrophil membrane proteins mediate the homing of nanoparticles to inflamed sites, and the FH peptide induces specificity because of its high affinity for tenascin‐C, which is produced by cardiac fibroblasts. The targeted delivery of miRNAs led to successful in vivo reprogramming, resulting in improved cardiac function and attenuation of fibrosis. Similarly, Wang et al. [182] used LNPs loaded with circular RNA of Tbx5 and siRNA against Ybx1 targeting cardiac fibroblasts for direct in vivo reprogramming to cardiomyocytes. The functional effect of LNP‐mediated direct reprogramming was evident from the improved cardiac function and reduced fibrosis following myocardial infarction.

### 4.4 Exosome‐Based Delivery

Exosomes are excellent delivery candidates because of their physicochemical stability, small size for deep tissue penetration [183], negative zeta potential for extended circulation time [184], and a lipid membrane that resembles the cell membrane [185]. Furthermore, the ease of engineering exosomes and loading different kinds of biologics makes them an attractive candidate for the in vivo delivery of the reprogramming factors [186, 187]. (Table 2).

The versatility of exosomes in cargo delivery makes them an ideal platform for potentiating additional functionality by combining them with exogenous molecules. Several approaches have been adopted to functionalize exosomes for the delivery of biologics and to generate therapeutic outcomes [188, 189]. One of the simplest methods to load cargo, albeit with low efficiency, is direct incubation with exosomes or exosome‐secreting cells [190, 191]. Physical approaches, such as sonication, extrusion, membrane permeabilization, and electroporation, have been designed to enhance the loading efficiency of biologics such as small molecules, proteins, and nucleic acids [189, 192, 193]. However, these approaches have limitations such as compromised exosomal membrane integrity and cargo aggregation, which affect cargo efficacy [188, 194, 195]. As an alternative, transfection of the cell of origin is extensively used for the stable loading of nucleic acids, peptides, and proteins into exosomes [188]. In this approach, producer cells are transfected with plasmids containing either coding or non‐coding RNAs under the regulation of strong promoters, such as the cytomegalovirus (CMV promoter) [196]. Higher cytoplasmic concentrations of these molecules lead to their loading into exosomes during biogenesis [197]. Once exosomes reach target cells, they release their cargo via direct membrane fusion or internalization via dynamin‐dependent or dynamin‐independent endocytic pathways [198, 199, 200].

Luo et al. [201] attempted to develop engineered exosomes for the delivery of a CRISPR‐Cas9‐based transcriptional activator system for target‐specific activation of Hnf4α in hepatic stellate cells (HSCs), thereby inducing a hepatocyte‐like phenotype. Infection of the AML12 hepatic cell line with plasmid vectors expressing dCas9‐VP64 (a transcriptional activator) and sgRNA against Hnf4α resulted in the release of exosomes carrying the plasmid as cargo. When injected via the tail vein of mice, exosomes accumulate in the liver and lungs, with increased mRNA and protein levels of Hnf4α in the liver.

### 4.5 Direct Administration

Direct administration involves the direct delivery of reprogramming factors in their native form without the use of any specific carriers into the bloodstream, relying primarily on passive diffusion and natural biodistribution for cellular uptake. Direct administration of reprogramming factors is either systemic, where the reprogramming factors are introduced into the circulation, or local, where they are delivered at the target site [202, 203, 204]. This approach is often employed for the in vivo delivery of small molecules because of the ease of administration, reduced immunogenicity, short‐term effect, and ease of multiple administrations (Table 2) [205, 206]. Huang et al. [207] systemically delivered a cocktail of small molecules designed to reprogram adult cardiac fibroblasts into cardiomyocytes. Of the seven molecules constituting the cocktail, five were administered orally, and two were delivered intraperitoneally. This systemic delivery of molecules led to in vivo reprogramming, albeit with a low conversion efficiency of 1%. The systemic delivery of small molecules for in vivo reprogramming is challenged by target specificity and reduced exposure of target cells to these small molecules. The direct administration of small molecules to the target organ is an approach to overcome these hurdles. One example of local administration of a small‐molecule cocktail was demonstrated by Yin et al. [80], who intracranially injected a cocktail of four small molecules to convert astrocytes into neurons. A single intracranial injection generated new neurons within 7 days and achieved a higher conversion efficiency than intraperitoneal injection, which required repeated administration over 3 weeks. Similarly, Ma et al. [208] used an osmotic mini‐pumping system for the localized and sustained delivery of a small‐molecule cocktail for the in vivo conversion of astrocytes to neurons. Microneedles have recently gained attention as innovative transdermal platforms for targeted and controlled drug delivery. Kannappan et al. [209] developed a microneedle patch loaded with nanoparticles encapsulating small molecules with the potential to induce the conversion of white adipocytes to brown adipocytes.

## 5 Therapeutic Application of In Vivo Reprogramming

The identification of new reprogramming factors and their successful delivery have led to the development of multiple protocols for reprogramming cells in vivo for regenerative medicine. These in vivo reprogrammed cells have the potential to replace cells lost following tissue injury in vivo and restore tissue functionality (Table 3). In the following section, we present examples of successful in vivo reprogramming aimed at therapeutic application and discuss how the inherent plasticity between lineage‐related cells can be leveraged to restore the function of damaged tissues.

**TABLE 3:** Representative examples of in vivo reprogramming.

Sl. No	Target organ	Cell conversion	Reprogramming factor	Delivery vehicle	Functional effect
1	Brain [210]	Glial cells to neurons	Transcription factors (Ascl1, LMX1a, and Nurr1)	Adeno‐associated viral vector	Functional neurons integrating with host neural circuit in 3 months
2	Brain [119]	Astrocytes to neurons	shRNA (against PTB)	Adeno‐associated viral vector	Replenishment of dopaminergic neurons and dopamine in Parkinson's disease model in 3 months
3	Heart [65]	Cardiac fibroblasts to cardiomyocytes	Transcription factors (Gata4, Mef2c, and Tbx5)	Retroviral vector	Improved cardiac function post‐myocardial infarction with functional cardiomyocyte integration in 3 months
4	Heart [106]	Cardiac fibroblasts to cardiomyocytes	miRNAs (miRNAs 1, 133, 208, and 499)	Lentiviral vector	Improved cardiac function after myocardial infarction in 3 months
5	Heart [207]	Cardiac fibroblasts to cardiomyocytes	Small molecules (CHIR99021, RepSox, Forskolin, VPA, Parnate, TTNPB, and Rolipram)	Oral (CRFTM) and intraperitoneal injection (VP)	Improved cardiac function after myocardial infarction in 1.5 months
6	Pancreas [143]	Pancreatic exocrine cells to beta cells	Transcription factors (Ngn3, Pdx1, and Mafa)	Adenoviral vector	Local angiogenesis, increased glucose tolerance, and serum insulin levels in 3 months
7	Adipose tissue [225]	M1 macrophages to M2 macrophages	Exosomes (ADSC‐derived exosomes)	Intraperitoneal injection	Reduction in white adipose tissue inflammation and improvement in insulin sensitivity in high‐fat diet‐fed mice in 2 months
8	Adipose tissue [209]	White adipocytes to brown adipocytes	Small molecules (SB431542 and NKH477)	Microneedle and nanoparticle	Improved energy expenditure, cold tolerance, and glucose sensitivity in high‐fat diet‐fed mice in 1 month
9	Eye [290]	Rod cells into cone‐like cells	Genome Editing by CRISPR/Cas9 (*Nrl* deletion)	Adeno‐associated viral vector	Improved visual function in retinitis pigmentosa mouse model in 1.5 months

### 5.1 Neurons

In vivo direct reprogramming to generate functional neurons from glial cells offers an opportunity to replace lost neurons and rebuild synaptic networks during neurodegeneration. In one of the earliest reports of in vivo neuronal reprogramming, Torper et al. [210] used Cre‐recombinase‐dependent AAV vectors to deliver a combination of three TFs, Ascl1, Lmx1a, and Nurr1 (ALN), for the direct conversion of NG2‐expressing glial cells into functional neurons in the striatum (Table 3) (Figure 3I). Injection of a viral vector carrying reprogramming factors led to the induction of a significantly high number of newly reprogrammed neurons, which could be analyzed for their phenotypic and functional properties in the long term after injury.

**FIGURE 3:**
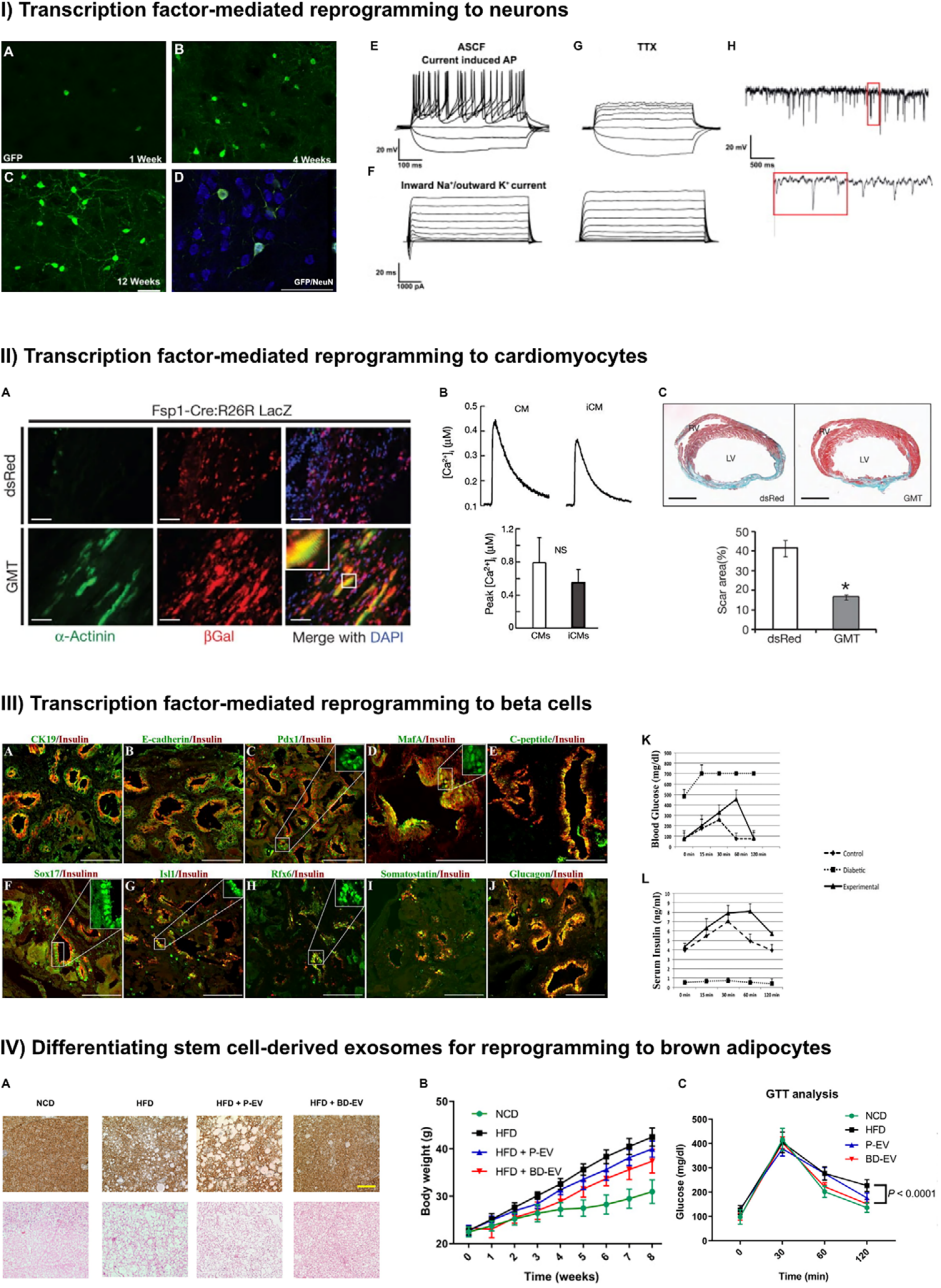
Representative examples of direct in vivo reprogramming. Sub‐figures labelled I), II), III), IV) denote different studies, and the letters denote individual panels. I) Transcription factor‐mediated reprogramming to neurons [210]. (A–C) Reprogramming of glial cells carrying GFP under the neuron‐specific synapsin promoter by transcription factors Ascl1, LMX1a, and Nurr1 (ALN) at different time points. Scale bar, 50 µm. (D) Expression of a neuronal and mature marker, NeuN (blue), in GFP^+^ neurons after 4 weeks of ALN administration. Scale bar, 25 µm. (E) Action potential (AP) of GFP^+^ neurons in response to depolarizing current. (F) Inward sodium (Na^+^) and outward potassium (K^+^) currents of GFP^+^ neurons upon depolarization. (G) Neurotoxin TTX‐mediated blocking of the AP, inward and outward currents of the reprogrammed neurons. Reproduced with permission from Torper et al. [210]. Copyright 2015 Elsevier. II) Transcription factor‐mediated reprogramming to cardiomyocytes [214]. (A) Representative fluorescence images in the infarcted area of dsRed‐ or GMT‐injected Fsp1‐Cre:R26R‐lacZ mouse hearts 4 weeks after infarction. α‐Actinin (green) labels cardiomyocytes, β‐galactosidase (βGal; red) marks Fsp1‐lineage cells, and DAPI (blue) represents nuclei. Scale bar: 50 µm. (B) Representative single‐field stimulated intracellular calcium (Ca^2+^) transients recorded from endogenous cardiomyocytes and GMT‐reprogrammed cardiomyocytes, showing comparable functional properties. (C) Masson‐Trichrome staining for scar quantification 8 weeks post‐infarction in dsRed‐ or GMT‐injected Fsp1‐Cre:R26R‐lacZ mouse. Scale bar, 500 µm. Reproduced with permission from Qian et al. [214]. Copyright 2012, Springer Nature Limited. III) Transcription factor‐mediated reprogramming to beta cells [216]. The delivery of transcription factors Pdx1, Ngn3, and MafA (PNM) generated insulin‐positive duct‐like cells expressing (A, B) epithelium markers CK19 and E‐cadherin, (C–H) beta cell markers PDX1, MAFA, C‐peptide, SOX17, ISL1, and RFX6, (I, J) pancreatic endocrine hormones glucagon and somatostatin. Scale bar, 1000 µm. (K) Glucose challenge showing better glucose disposal of PNM‐treated diabetic mice compared to untreated diabetic mice. (L) Glucose‐responsive elevation in serum insulin in PNM‐treated mice. Reproduced with permission from Banga et al. [216]. Copyright 2012, National Academy of Sciences. IV) Differentiating stem cell‐derived exosomes for reprogramming to brown adipocytes [229]. (A) Representative images of UCP1 (top) and H&E (bottom) staining of interscapular brown adipose tissue from normal chow diet (NCD)‐fed, high‐fat diet (HFD)‐fed mice, and HFD‐fed mice treated with exosomes isolated from proliferating ADSCs (HFD + P‐EV), and beige adipocyte‐differentiating ADSCs (HFD + BD‐EV). Scale bar, 100 µm. (B) Body weight change over 8 weeks in NCD‐fed mice and HFD‐fed mice administered P‐EV and BD‐EV twice a week. (C) Glucose tolerance test in NCD‐fed mice and HFD‐fed mice administered P‐EV and BD‐EV, showing improved glucose metabolism in BD‐EV‐treated groups. Reproduced with permission from Jung et al. [229]. Copyright 2020, The American Association for the Advancement of Science.

Qian et al. [119] used an AAV vector for the expression of shRNA against PTB (AAV‐shPTB) for direct in vivo reprogramming of mouse astrocytes into neurons (Table 3). Lineage tracing experiments demonstrated that 3 weeks after AAV‐shPTB injection, the reprogramming efficiency was approximately 20% (based on NeuN expression), which gradually increased to 80% of NeuN^+^/Gfap^−^ cells 12 weeks after injection. After 12 weeks, the newly generated neurons showed typical voltage‐dependent currents in the sodium and potassium channels, repetitive action potential firing, and spontaneous postsynaptic currents. Interestingly, the commitment of neurons to a specific subtype was dependent on the specific area of the AAV‐shPTB injection. The midbrain contained dopaminergic neurons, whereas the majority of reprogrammed cells in the cortex expressed markers of cortical neurons. This observation is consistent with previous reports of differential gene expression patterns associated with astrocytes obtained from different regions of the brain [211]. The basal expression levels of TFs regulating dopaminergic fate were higher in astrocytes from the midbrain than in those from the cortex, which, upon PTB suppression, drives neural reprogramming toward a specific fate. In a Parkinson's disease model, injection of AAV‐shPTB replenished the lost DA neuronal density by 30% and striatal dopamine by 65%, reversing disease‐relevant motor phenotypes within 3 months of injection.

### 5.2 Cardiomyocytes

Cardiac fibroblasts serve as the starting cells for cardiomyocyte reprogramming because of their abundance and role in cardiac injury [212, 213]. One of the earliest examples of in vivo cardiac reprogramming was reported by Qian et al. [214], who used retrovirus‐mediated delivery of three TFs, Gata4, Mef2c, and Tbx5, for the direct reprogramming of cardiac fibroblasts into cardiomyocytes (Table 3) (Figure 3II). The newly induced cardiomyocytes became binucleated and developed mature sarcomeres four weeks after the delivery of the reprogramming factors. Furthermore, these cells exhibit action potentials, beating patterns, and electrical coupling similar to those of endogenous cardiomyocytes. The simultaneous delivery of a proangiogenic and fibroblast‐activating peptide, thymosin β4, resulted in reduced fibrosis and improved cardiac function. Interestingly, the newly induced cardiomyocytes were fully reprogrammed, in contrast to their in vitro counterparts, and exhibited molecular signatures similar to those of endogenous cells.

In an in vitro setting, Jayawardena et al. [106] reported a combination of four miRNAs (1, 133, 208, and 499) (miR combo) with the potential to reprogram cardiac fibroblasts into functional cardiomyocytes in vitro. The combination of miRNAs had a synergistic effect in inducing successful reprogramming trajectories, leading to the generation of cardiomyocytes. The lineage‐tracing approach showed that the newly reprogrammed cardiomyocytes incorporated into the surrounding cardiac syncytium constituted 1% of the total cardiomyocyte population after six weeks of miR combo administration. In a follow‐up study, Jayawardena et al. [215] showed that in vivo newly reprogrammed cells exhibit structural and functional features of mature ventricular cardiomyocytes 5–6 weeks post‐infarction (Table 3). In vivo reprogramming of non‐cardiomyocytes into cardiomyocytes resulted in functional recovery in infarcted mice within 3 months.

Similarly, Huang et al. [207] reported small molecule‐mediated cardiac reprogramming following myocardial infarction, leading to functional recovery. Based on an in vitro protocol, Huang et al. [207] attempted to deliver the cocktail CRFVPTM (C, CHIR99021; R, RepSox; F, Forskolin; V, VPA; P, Parnate; T, TTNPB; M, Rolipram) for the in vivo conversion of cardiac fibroblasts to cardiomyocytes (via oral administration of CRFTM and intraperitoneal injection of VP) (Table 3). Administration of the cocktail once a week for six weeks generated long rod‐shaped cells showing key cardiomyocyte‐specific markers and well‐developed sarcomere structures with an efficiency of up to 1%, leading to functional improvement of the cardiac tissue. The chemically induced cardiomyocytes, coupled with endogenous cardiomyocytes, generated action potentials similar to those generated by the atrial cardiomyocytes.

### 5.3 Pancreatic and Liver Cells

A mutation study of the TFs involved in the differentiation of beta cells identified a combination of three TFs, Ngn3, Pdx1, and Mafa, which could reprogram more than 20% of the infected cells into insulin^+^ cells 10 days post‐viral delivery (Table 3) [143]. Reprogrammed beta cells were functional and secreted insulin and vascular endothelial growth factors that induced local angiogenesis. In a diabetic mouse model, the delivery of these TFs increased glucose tolerance and serum insulin levels. Interestingly, the transient expression of these reprogramming factors was sufficient to induce insulin^+^ cells in the long term in mice.

Because pancreatic and liver cells are closely related owing to their common progenitors, the successful reprogramming of pancreatic cells has motivated attempts to reprogram liver cells. In one of the first attempts, Banga et al. [216] reported in vivo conversion of Sox9^+^ cells present in the liver to insulin‐secreting ducts in immunodeficient mice using the TFs Pdx1, Ngn3, and MafA (Figure 3III). The insulin^+^ and C‐peptide^+^ cells generated were also found to release other hormones that are not specific to beta cells, such as glucagon and somatostatin, and were stable even after suppression of the initiating factors. Interestingly, these insulin‐secreting cells exhibited phenotypic similarities with duct‐like structures and expressed non‐beta cell‐specific cholangiocyte markers, such as CK19 and E‐cadherin. These features suggest the involvement of a novel, uncharacterized cell state with properties distinct from those of any defined progenitor or terminally differentiated cells. Lineage‐tracing experiments demonstrated that Sox9^+^ cells present in the biliary tract are the main targets of the reprogramming factors used. During liver development, bipotent hepatoblasts give rise to Sox9^+^ cholangiocyte precursors that form the ductal plate, eventually resulting in primitive ductal structures [216, 217]. Owing to the relatively plastic characteristics of progenitors and a common progenitor, the molecular signatures of Sox9^+^ cells may facilitate the activation of pancreatic‐associated GRN upon exposure to an appropriate reprogramming factor [218]. However, a detailed mechanistic study is required to determine the GRN targeted by reprogramming factors and how they might lead to the induction of mature cholangiocyte markers.

### 5.4 Adipocytes

Given the ability of brown and beige (brown‐like) adipocytes to dissipate energy, improve metabolic health, and regulate obesity, promotion of browning or beiging of white adipose tissue (WAT) represents a promising strategy for combating obesity and insulin resistance [219, 220, 221, 222, 223, 224]. To this end, Zhao et al. [225] showed that intraperitoneal administration of ADSC‐derived exosomes could lead to polarization of M1 macrophages toward an anti‐inflammatory M2 phenotype and induce beiging in WAT (Table 3). This polarization led to a 27% reduction in WAT inflammation and improvement in insulin sensitivity. In addition to immunomodulation, ADSC‐derived exosomes promote WAT beiging and facilitate energy expenditure during a long‐term high‐fat diet regimen. Exosome‐induced M2 macrophages produce high levels of tyrosine hydroxylase and lactate, thereby favoring WAT beiging [226, 227, 228]. Similarly, Jung et al. [229] used exosomes derived from ADSCs undergoing beige adipogenic differentiation to ameliorate diet‐induced obesity and hepatic steatosis, and improved glucose metabolism by inducing adipose‐tissue browning (Figure 3IV). The human adipokine array showed the presence of multiple adipokines, such as adiponectin, adipsin, and angiopoietin‐like 4, in exosomes, which are known to play key roles in adipogenesis. miRNA expression profiling showed that miRNAs that positively regulate brown fat cell differentiation, such as miR‐193b, miR‐196a, miR‐328, and miR‐378a, were significantly enriched. Furthermore, pathway enrichment analysis showed that the target genes of the enriched miRNAs were associated with the MAPK, Ras, and ErbB signaling pathways, highlighting that exosomes carrying a subset of biologics activate a GRN that governs cell fate.

## 6 Challenges in Bench‐to‐Bed Translation

In vivo direct reprogramming has shown tremendous potential for the functional restoration of organs without the need for whole‐organ transplantation (Table 3). However, despite the development of numerous reprogramming systems and encouraging results from preclinical studies, in vivo direct reprogramming has not been able to make it to clinical trials yet. The therapeutic outcomes of in vivo reprogramming are often hindered by the complex 3D dynamic tissue environment, which is challenging to replicate in an in vitro system. The tissue microenvironment also provides biophysical and biochemical cues, capable of integrating with the molecular rewiring process, which are often overlooked in an in vitro setting. Moreover, the lack of a proper mechanistic understanding of direct reprogramming raises safety concerns regarding the administration of reprogramming factors in clinical settings. Here, we briefly review the bottlenecks that hinder the application of in vivo direct reprogramming in a clinical setting.

### 6.1 Kinetics and Efficiency of In Vivo Direct Reprogramming

One of the major limitations of in vivo reprogramming in terms of generating therapeutic outcomes is the disproportionate dose and exposure time of reprogramming factors to the target cells, which results in slow reprogramming kinetics and poor efficiency. Although in vitro direct reprogramming systems have optimized the dose and exposure time for successful conversion, reprogramming factors in vivo are challenged by the dynamic and complex 3D tissue microenvironment, which shields target cells from the reprogramming factors. It has been reported that the in vitro‐optimized reprogramming factors’ dose or exposure time is insufficient for successful in vivo direct reprogramming. In an in vivo study, the in vitro‐optimized concentrations of small molecules were found to be insufficient to bring about a change in cell fate, and a concentration as high as three times was found to be effective to reprogram cells [208]. In addition to an appropriate dose, an appropriate exposure time for reprogramming factors is required to generate a stable cell state and prevent the generation of partially or non‐reprogrammed cells [230, 231]. Partially reprogrammed cells can either return to their original state or possess the potential to generate cells from different or related lineages upon receiving biological cues from the tissue microenvironment, which could be a concern in a complex tissue environment [19, 232, 233, 234]. In contrast to the short exposure time, the extended and sustained presence of reprogramming factors is undesirable, as it may impart modified or additional characteristics to the cells. Consequently, repeated administration of reprogramming factors is often required to maintain an appropriate exposure time and generate stable cell states. For instance, for the in vivo chemical reprogramming of astrocytes to neurons, Ma et al. [208] continuously administered a cocktail in the brain of an adult mouse for two weeks, which generated NeuN^+^ cells with an efficiency of approximately 11.3% eight weeks after induction. Notably, when the same cocktail was applied in vitro, neonatal mouse astrocytes were successfully reprogrammed into NeuN^+^ neurons with an efficiency of 94.1% within four weeks. This stark contrast underscores a key limitation: the absence of an in vitro system that accurately replicates the complex, 3D, and dynamic tissue microenvironment, which hampers the preclinical optimization of reprogramming protocols, together posing a significant barrier to the efficient generation of therapeutically relevant cells in vivo.

Although new delivery approaches are being adopted, the local delivery of reprogramming factors in a controlled and sustained manner remains challenging. In addition, the route of administration of reprogramming factors warrants further investigation, as it critically influences tissue exposure and may significantly enhance reprogramming efficacy. In a related study on small molecules‐mediated astrocyte‐to‐neuron conversion, a comparison between intracranial and intraperitoneal administration established the superiority of the former, thus highlighting the significance of route of administration in in vivo reprogramming [80]. Given the prolonged process required for in vivo reprogramming, the circulation time and half‐life of reprogramming factors are critical parameters that warrant careful evaluation, and strategies are required to enhance their stability and bioavailability. Moreover, to achieve controlled and sustained local delivery, it is essential to optimize the composition of the reprogramming cocktail, potentially by minimizing the number of components to enable precise regulation of release kinetics.

### 6.2 Biophysical and Biochemical Cues Present In Vivo

The influence of tissue microenvironment, including cell‐cell and cell‐matrix interactions, and local biochemical cues, on the efficacy of reprogramming factors remains insufficiently understood and warrants further investigation (Figure 4A). Indeed, the presence of lineage‐related cells and tissue‐specific niches promotes functional maturation of cells and results in better reprogramming than their in vitro counterparts [80, 214]. However, it would be interesting to investigate whether tissue‐resident biophysical and biochemical cues antagonize the effects of reprogramming factors and hamper their efficacy and specificity in vivo. The biophysical cues present in the tissue microenvironment include fluid‐flow‐induced shear stress, the mechanical and electrical properties of ECM, among others [235, 236]. These properties are well known to affect functionality and maturation of cells [235, 237]. The biophysical cues are sensed by integrins present on the surface of cells and eventually translated into signaling pathways and transcriptional and epigenetic regulators [236, 238, 239]. The failure of in vitro direct reprogramming systems to recapitulate biophysical cues leads to cultured cells adopting a molecular state distinct from their in vivo counterparts, which could possibly affect the reprogramming outcome [240, 241]. Another aspect that needs to be considered for tissue regeneration following injury is the cascade of biochemical cues that are activated as endogenous reparative responses. As reprogramming factors are known to activate certain GRNs and are selective to the initial cell state, it is possible that injury‐induced molecular events modulate the cell state and, therefore, the reprogramming process [242, 243, 244, 245, 246]. On this note, Chen et al. [247] demonstrated that in vivo Ptbp1 suppression in reactive astrocytes failed to generate dopaminergic neurons in a mouse model of Parkinson's. Another aftereffect of tissue injury is the generation of reactive oxygen species by polymorphonuclear neutrophils, which facilitates the migration of inflammatory cells to the injury site [248]. The subsequent induction of redox signaling and oxidative stress affects both cellular physiology and pathology, which can plausibly affect direct reprogramming [249, 250]. Indeed, in an attempt to convert astrocytes into neurons, Russo et al. [251] reported that introducing ROS‐scavenging molecules reduced reprogramming efficiency, highlighting the important role of ROS in facilitating cellular fate changes. It is also important to note the effects of cellular senescence induced by tissue injury. Interestingly, cellular senescence has been reported to negatively impact in vitro direct reprogramming but promote reprogramming in vivo by enhancing tissue plasticity and regeneration [252, 253, 254, 255]. These observations underscore the need to employ disease‐relevant models to optimize and refine reprogramming strategies under pathological conditions. Another challenge in in vivo direct reprogramming is the specific targeting of cells in a complex tissue environment, in which different cell types are spatially arranged to impart organ functionality (Figure 4A). Unintended exposure of non‐target cells to reprogramming factors may pose theoretical risks to organ function owing to cell identity‐specific molecular signatures.

**FIGURE 4:**
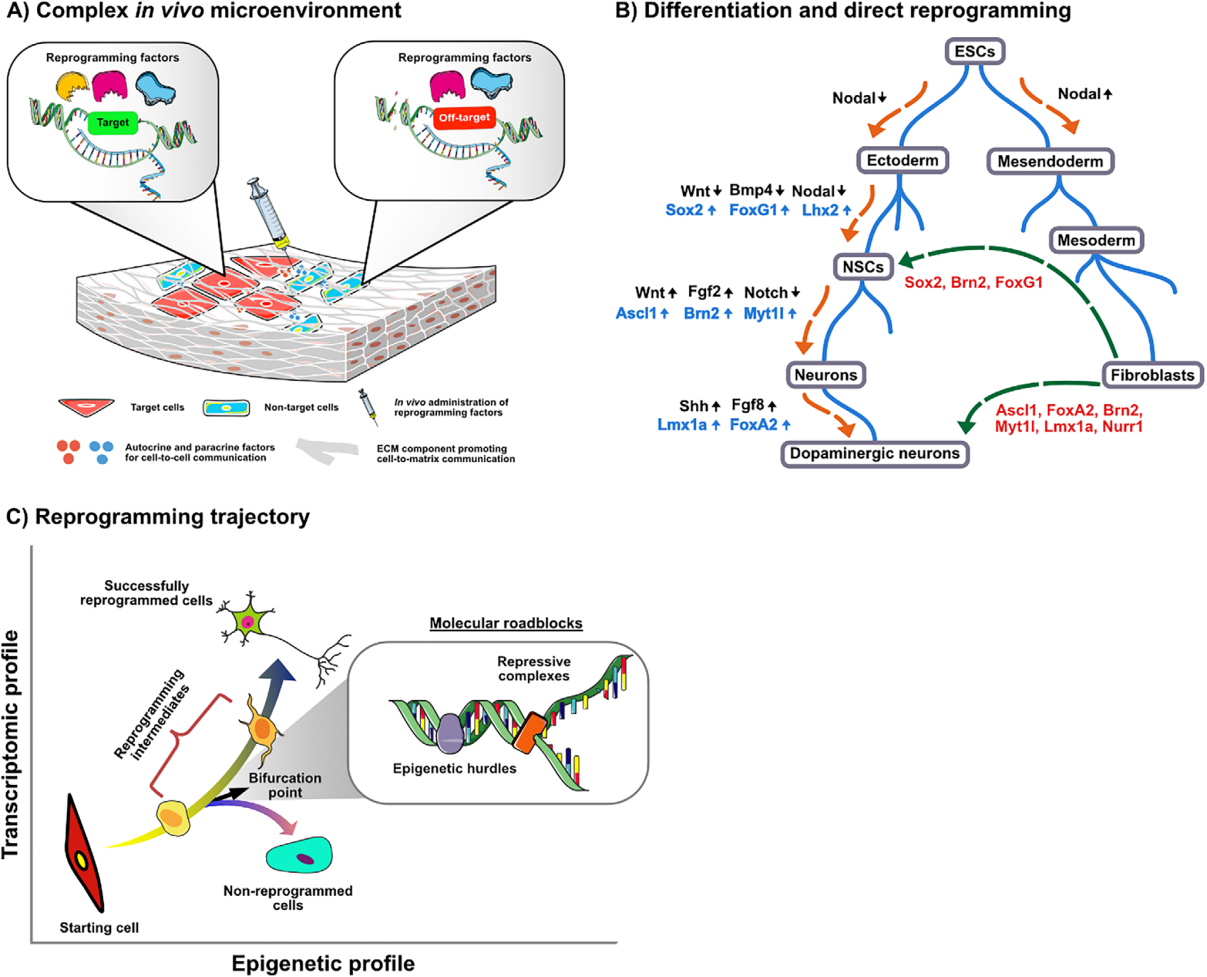
(A) 3D tissue microenvironment showing target and non‐target cells embedded within the tissue matrix that act as a biophysical cue. As shown in the figure, the cells release various autocrine and paracrine factors within the microenvironment, which influence cellular behavior and may potentially interfere with the reprogramming process induced by the exogenous reprogramming factors. After administration, the reprogramming factors also affect non‐target cells, which may respond differently from the intended target cells, leading to non‐specific and potentially undesirable effects. (B) The differentiation trajectory of ESCs to different lineages and key components of various GRNs governing the differentiation during development. The orange line on the left shows the differentiation of ESCs to dopaminergic neurons through different developmental stages. Texts in the black font represent the signaling pathways that regulate the generation of the following developmental stages, with arrows representing their upregulation or downregulation. Texts in the blue font represent the key transcription factors that serve as master regulators at each developmental stage. The schematic also shows the direct reprogramming of fibroblasts to different developmental stages of the neural lineage in the presence of various reprogramming factors, depending on which stage of the GRN is targeted. The treatment of fibroblasts with transcription factors such as SOX2, BRN2, and FOXG1, which are known to regulate the generation of NSCs, results in direct reprogramming to NSCs. Similarly, the use of transcription factors ASCL1, LMX1A, NURR1, BRN2, FOXA2, and MYTL1 directly reprogram fibroblasts to dopaminergic neurons because of the known role of LMX1A and FOXA2 in the specification of neurons to the dopaminergic subtype. (C) Reprogramming trajectory showing the transition in the transcriptomic and epigenetic profile and various reprogramming intermediates involved during the reprogramming process. The schematic shows two different reprogramming trajectories emerging at the bifurcation points, leading to successfully reprogrammed cells and non‐reprogrammed cells, respectively. The bifurcation of the trajectories is governed by certain molecular roadblocks controlling the epigenetic or transcriptomic signatures that restrict successful reprogramming. In a heterogeneous cell population undergoing reprogramming, cellular states that can overcome the molecular hurdles lead to the generation of successfully reprogrammed cells. Figures were drawn using pictures from Servier Medical Art, by Servier, licensed under a Creative Commons Attribution 4.0 Unported License. Figures were designed or edited using Affinity Designer, a vector graphics software, to create high‐resolution vector illustrations and infographics.

### 6.3 Mechanistic Understanding

In addition to the precise delivery of reprogramming factors, the lack of a comprehensive mechanistic understanding of ‘direct reprogramming’ raises safety concerns regarding its clinical application. The TFs used for direct reprogramming are well‐known pioneering factors that activate GRNs, thereby governing the generation of a specific cell state [21]. The developmental hierarchy of these GRNs dictates reprogramming trajectories, which often involve cell states with molecular signatures resembling those of predefined developmental progenitors [256]. These progenitor stages have the potential to generate lineage‐related cells that are non‐specific to a particular tissue microenvironment. For example, directed reprogramming of fibroblasts using the TFs FoxA1 and Hnf4a generates endodermal progenitor cells with the ability to integrate into both the liver and intestine [257]. The emergence of progenitor‐like molecular signatures during reprogramming highlights the need to understand the extent of ‘direct reprogramming’, particularly the involvement of intermediate cell states and hierarchical levels of GRNs in the process. Additionally, the frequent overlap of small molecules across protocols targeting different lineages highlights the importance of mapping and characterizing reprogramming trajectories and intermediates to minimize the risk of generating non‐specific or undesired cell types in vivo. For instance, small molecules, such as CHIR99021, Forskolin, and RepSox, which target the WNT, cAMP, and TGF‐β pathways, respectively, are commonly included in reprogramming cocktails to induce pluripotent stem cells, cardiomyocytes, hepatocytes, and neurons, despite their distinct developmental origins [63, 80, 207, 258].

A better understanding of the reprogramming trajectory can lead to the refinement of reprogramming factors to generate lineage‐restricted cells by specifically targeting GRNs associated with relatively lower levels of developmental hierarchy (Figure 4B). Furthermore, knowledge of the GRN involved in the functional maturation of cells can help screen for additional factors that can generate therapeutically functional cells [256]. One of the critical factors for successful reprogramming is the appropriate selection and exposure time of reprogramming factors to prevent the generation of partially reprogrammed cells or cellular reversion to the original state. During in vitro direct reprogramming of fibroblasts to induce neuron‐like cells, Gary et al. [259] reported that extended exposure to the epigenetic modifier JQ‐1(+) is required to maintain the induced neuronal program and prevent reversion. In this regard, the application of single‐cell technologies in direct reprogramming can facilitate the identification of bifurcation points of reprogramming trajectories and the key molecular players responsible for successfully separating reprogrammed cells from partially or non‐reprogrammed cells (Box 2) (Figure 4C) [260].

**Box 2: Single‐cell Informatics**

Single‐cell informatics lies at the intersection of single‐cell multi‐omics technologies and computational methods, aiming to define and characterize the state of individual cells based on their epigenome, transcriptome, proteome, and metabolome. As heterogeneous cell populations emerge during processes of biological transitions or direct reprogramming, understanding the distinct cellular states and identifying the molecular regulators at various stages becomes essential.

**Dimensionality Reduction**: Dimensionality reduction is the process of simplifying the high‐dimensional data generated from the omics analysis. This process reduces the data to two or three dimensions while preserving both local and global structures, thereby enabling the visualization of meaningful clusters that reflect underlying biological relationships.

**Trajectory Inference**: Trajectory inference reconstructs the continuous biological progression through which cells transition from one state to another. Each point along the inferred trajectory corresponds to the molecular state of a single cell. The nature of the trajectory, linear or branched, depends on whether cells converge to a single fate or diverge into multiple lineages during the process.

**Pseudotime Analysis**: Pseudotime analysis assigns cells a value along a continuum that reflects their relative position within a dynamic process, based on their molecular features. This computational ordering facilitates the identification of transitional or rare intermediate states, branching points, and key regulatory factors driving these shifts.

**Lineage Tracing**: Lineage tracing is an experimental strategy used to map the ancestral relationships between cells during development or other dynamic processes. It enables the tracking of clonal histories within a heterogeneous population and uncovers the molecular cues influencing these trajectories.

In direct reprogramming, single‐cell informatics helps in identifying the trajectories associated with successful and unsuccessful reprogramming and the molecular players governing them. Furthermore, it also helps in characterizing the reprogramming intermediates and identifying the cellular states present in a heterogeneous population that would result in successful reprogramming.

### 6.4 Safety Concerns

One of the major challenges in in vivo direct reprogramming is the inability to target a specific cell type within the tissue. The safety concerns associated with the non‐specific targeting remain undefined, as in vitro‐based protocols do not often validate the effect of reprogramming factors on non‐target cells. Since tissue‐resident cells typically originate from a common progenitor and exhibit molecular similarity to some extent, the non‐target cells are often susceptible to the reprogramming factors, which can potentially alter their cellular state or functionality [30, 31, 261, 262]. This alteration can disrupt tissue architecture and functionality by changing the properties of different cell types within the tissue [263, 264]. In addition to this, many of the reprogramming factors induce a progenitor‐like state in the cells, which raises concerns regarding their non‐specific differentiation under the influence of tissue‐resident biophysical and biochemical cues [257, 265, 266].

Another safety concern associated with in vivo direct reprogramming is the tumorigenesis risk associated with the various reprogramming factors and delivery vehicles. The use of reprogramming factors with oncogenic potential such as c‐Myc and Sox2, and epigenetic modifiers which can induce widespread epigenetic remodelling and silence tumor suppressor genes or derepress oncogenes, poses a risk of tumor formation [267, 268]. Moreover, the induction of a transient progenitor or dedifferentiated state in the tissue microenvironment during in vivo direct reprogramming can mimic a cancer‐like phenotype [269, 270, 271, 272]. The inability of in vitro systems to replicate the tissue‐resident biochemical cues and study their effect on the transient cellular states makes it difficult to predict their fate. In addition to this, the use of integrating vectors for the delivery of reprogramming factors is associated with the increased risk of insertional mutagenesis [22]. Since such vectors contain a strong promoter and the integration is not controlled, they can potentially alter the chromatin arrangement and expression patterns of genes, including oncogenes and tumor suppressors [273, 274, 275, 276].

The use of integrating vectors for the delivery of reprogramming factors leads to their extended and sustained presence, which may impart modified or additional characteristics to the cells. For instance, the application of integrating vectors to deliver the reprogramming factor Ascl1 results in a fate change from astrocytes to neurons [22, 47]. However, because this reprogramming TF functions as a pioneer factor and activates GRNs that are different from those present in terminally differentiated neuronal cells, its genomic integration and continued expression may result in undesirable gene expression, with unknown and undefined challenges yet to be characterized. Although other approaches, such as the use of non‐integrating vectors, plasmids, and small molecules, bypass the risk of continued exposure, their reprogramming efficacy is often hindered by the limitations associated with local delivery and the low shelf life of reprogramming factors, which could potentially lead to incomplete reprogramming [30, 277].

It would also be interesting to see if in vivo reprogrammed cells retain their identity and functionality after the short exposure to reprogramming factors. Since direct reprogramming approaches do not induce a complete resetting of the epigenome, the reprogrammed cells often possess the tendency to revert back to the original cell state, making the reprogramming unsuccessful and resulting in an altered cellular state that is not often characterised [278]. In particular, during in vivo reprogramming, the fate and characterization of such cells are of utmost importance to avoid any risk caused by an undefined cell state exhibiting enhanced or modified characteristics. Recent in vitro studies have investigated the effects of partial reprogramming on the transcriptomic and epigenetic states of cells. In iPSC‐based reprogramming systems, partially reprogrammed cells have been found to exhibit rejuvenated epigenetics resembling those of young cells, accompanied by the expression of developmental regulators, improved metabolism, and stress sensitivity [279, 280, 281]. These cells exhibit enhanced regenerative potential and plasticity and can possibly generate cells from other lineages when exposed to appropriate biochemical cues [282, 283]. Therefore, non‐reprogrammed and partially reprogrammed cells must be thoroughly investigated for changes in their molecular signatures due to exposure to reprogramming factors.

## 7 Future Directions

Over the past decade, in vivo direct reprogramming has emerged as a powerful strategy in regenerative medicine, offering the potential to convert resident cells into therapeutically functional cell types within their native microenvironment. Although significant progress has been made in identifying reprogramming factors and delivery methods, this field still faces several critical challenges and has failed to enter clinical trials. The main hurdles in the clinical translation of direct in vivo reprogramming include low reprogramming efficiency, insufficient mechanistic understanding, and difficulty in achieving cell type specificity in complex tissue contexts. Therefore, new strategies must be pursued and developed to overcome these issues to improve the therapeutic efficacy and motivate the efforts to initiate clinical trials of in vivo direct reprogramming.

In this section, we introduce emerging approaches such as the development of novel reprogramming factors, delivery approaches with precise targeting, in vitro platforms that mimic the tissue microenvironment, strategies to elucidate molecular mechanisms, and multimodal approaches favoring bench‐to‐bed translation.

### 7.1 Development of Novel Reprogramming Factors

The limitations of conventional reprogramming factors highlight the need for innovative strategies beyond traditional TFs and small‐molecule approaches. Recently, reprogramming factors such as metabolic regulators have gained attention for their ability to modulate stem cell fate in vivo and guide cells toward specific lineages to promote tissue regeneration. Chi et al. [284] showed that a bioactive lipid, 11,12‐EET, released from macrophages post‐injury, promotes the myogenesis of muscle stem cells and enhances muscle regeneration. Similarly, metabolic reprogramming of T cells using specific drugs or delivery of mRNA encoding chimeric antigen receptor T cells in vivo is yet another potential avenue that has shown potential to empower immune cells to fight against cancer cells [285, 286].

Synthetic biology offers an alternative approach for modulating cell fate by rewiring molecular signatures using synthetic transcriptional, epigenetic, or signaling pathway regulators [287]. The CRISPR/Cas9 system is a promising synthetic biological approach for regulating cell fate. CRISPRa/dCas9‐based transcriptional modulators have opened up new possibilities for the precise and programmable activation of lineage‐specific gene networks in vivo [288, 289]. Moreno et al. [290] used an AAV vector platform for the in vivo delivery of a CRISPR/Cas9 system designed to repress *Nrl*, facilitating the in situ reprogramming of rod cells into cone‐like cells (Table 3). Complementing this is the development of computational models that aid in the generation of artificial transcription factors (aTFs) for targeted gene expression to induce cell fate change [291, 292, 293]. These approaches inform aTF design by predicting and engineering effective DNA‐binding domains with defined motif preferences [294, 295, 296, 297]. The predicted DNA‐binding domains can be combined with various effector domains on different scaffolds and delivery vehicles (e.g., dCas9, zinc fingers, TALEs, nanoparticles) to generate functional artificial transcription factors [297, 298, 299, 300, 301, 302].

The convergence of artificial intelligence, machine learning, molecular biology, and advanced imaging techniques has enabled the integration of large‐scale omics and phenotypic data. These datasets can be exploited to predict gene perturbation effects, induction of GRNs, and cell state transitions for the identification of novel reprogramming factors [303, 304, 305]. Several models have been developed that can predict cell state transitions and the outcome of multigene or proteomic perturbations, potentially leading to the refinement of reprogramming strategies [306, 307, 308]. Furthermore, machine learning‐based models have been developed that exploit microscope‐based phenotypic transitions during direct reprogramming to predict the proportion of cells following the successful reprogramming trajectories [309, 310]. Such models can be used along with large‐scale perturbations, such as pooled CRISPR perturbations, to screen for a novel set of reprogramming factors.

Another aspect of in vivo direct reprogramming which warrant the refinement of reprogramming factors or identification of novel factors is the tissue‐resident biophysical cues [311, 312, 313]. These cues are well documented to activate epigenetic readers and writers and modulate the epigenetic and transcriptomic landscape [239, 266, 314]. Recently, Ha et al. demonstrated that Desmosomes, mechanosensitive intercellular junctions that sense and transmit tissue‐resident biophysical cues, regulate both in vitro direct reprogramming and regeneration in vivo [315]. Moreover, several studies have reported the modulation of substrate stiffness during in vitro direct reprogramming to improve the reprogramming efficiency [313, 316]. These studies underscore the importance of understanding how biophysical cues influence cellular molecular states and the need for reprogramming factor refinement accordingly. In this regard, computational frameworks that establish a causal relationship between tissue‐specific biophysical cues and the molecular signatures can provide a potential guideline for in vivo direct reprogramming. Recently, Vinayak et al. developed a sequencing‐informed polymer model that integrates ChIP‐seq and Hi‐C datasets to simulate the effects of biophysical cues on chromatin organization [317]. The model predicted heterochromatin domain boundaries to be the most susceptible genomic regions to mechanically‐induced epigenetic remodeling. Complementing this approach, Xu et al. developed a causal multi‐omics framework that integrates tissue‐resident biophysical cues with transcriptomic data to identify the GRN which are directly regulated by tissue‐induced mechanotransduction [318]. Development of such computational models can provide a framework for the rational design of in vivo direct reprogramming strategies taking into consideration the tissue‐resident biophysical cues and their role in modulating the molecular landscape of cells. Furthermore, such an understanding can also guide the development of strategies for the transient remodelling of tissue‐resident extracellular matrix, thereby inducing a permissive biophysical microenvironment that would facilitate direct in vivo reprogramming [313, 316].

### 7.2 Controlled and Targeted Delivery

Innovative approaches are emerging for the controlled and targeted delivery of reprogramming factors. Nanochanneled devices have been developed for cell‐specific targeting that use an intense, focused electric field to achieve controlled cytosolic delivery of reprogramming factors, a process referred to as tissue nanotransduction [319, 320]. Similarly, nanoparticle therapeutics consisting of various biologics encapsulated in different types of nanoparticles have been explored for tissue‐specific delivery of reprogramming factors [173]. Microneedle patches are used for noninvasive transdermal delivery of reprogramming factors to induce localized and targeted effects [321]. Hydrogels and polymeric implants have been exploited for the controlled and sustained release of several drugs, thereby improving their half‐life, biodistribution, and maintenance of therapeutic concentrations over extended periods [322, 323]. The application of such platforms for the targeted and controlled delivery of reprogramming factors holds significant promise for enhancing the efficacy of in vivo reprogramming techniques. In one such attempt, Iao et al. [324] developed a conducting microgel coated with a porous molybdenum carbide octahedron capable of releasing oxygen and electrical signaling in response to an external alternating magnetic field. The synergistic effect of oxygen release and electrical stimulation led to a rewiring of transcriptomic signatures, influencing biological processes such as angiogenesis, somatostatin and GABA production, that eventually led to regeneration and functional recovery following traumatic brain injury. In addition to these, external biophysical cues such as ultrasound, electromagnetic fields (EMFs), and irradiation can act as triggers to promote the controlled release of reprogramming factors or induce stimulus‐responsive molecular changes for in vivo direct reprogramming [325, 326, 327, 328, 329]. In one such attempt, Yoo et al. [330] reported the stimulation of magnetized gold nanoparticles using EMFs for facilitating in vivo direct neuronal reprogramming. Upon co‐transplanting these nanoparticles with lentiviral particles containing the reprogramming factors, EMF stimulation induced an epigenetic remodelling to create a transcriptionally permissive state and facilitate direct reprogramming. Similarly, Moon et al. [331] developed a microneedle‐based platform consisting of mesenchymal stem cell‐derived extracellular vesicles (EVs) and near‐infrared‐responsive MXene nanoparticles for the stimuli‐based targeted delivery of the extracellular vesicles, leading to macrophage polarization and wound healing. Recently, Kim et al. [332] reported an EMF‐responsive gene switch for controlled and precise spatiotemporal gene expression. The EMF exposure resulted in a Cyb5b‐mediated oscillatory Ca^2+^ influx, which eventually led to the rapid and reversible activation of transcriptional regulator Sp7. Using this EMF‐responsive gene switch, the authors showed the controlled activation of several genes by placing them under the control of an EMF‐inducible DNA element for in vivo direct reprogramming and functional manipulation.

### 7.3 Organ‐on‐Chips

Organ‐on‐chips are microphysiological systems, consisting of a microfluidic device mounted with a 3D culture system, that promise to replicate the tissue microenvironment and offer a promising platform for mimicking the in vivo microenvironment (Box 3) [333]. Microfluidic devices are designed to emulate the dynamic in vivo environment by incorporating micro‐engineered tissues and organoids cultivated in a 3D extracellular matrix (ECM) gel that mimics tissue‐specific cellular composition and biophysical and biochemical properties in the presence of dynamic fluid flow [334]. These devices have been successfully used to investigate the efficacy of reprogramming factors and are plausible platforms for optimizing the dose and treatment duration of reprogramming factors required in a 3D and dynamic setup [335, 336]. Recently, Kajtez et al. [337] demonstrated direct in vitro reprogramming of adult human fibroblasts into neurons within suspension 3D microculture arrays. These reprogrammed neurons exhibited improved cell viability and functionality compared to those obtained using traditional methods, highlighting the superiority of 3D‐based reprogramming over conventional reprogramming in a 2D setup. Similarly, small molecule‐induced direct cardiac reprogramming in heart‐derived cardiac ECM resulted in enhanced reprogramming with the generation of functionally mature cardiomyocytes [338]. The ability of microfluidic devices to mimic the in vivo biophysical cues such as fluid‐induced shear stress, matrix stiffness, and electrical properties of ECM can further help in refining reprogramming factors to generate mature cells, which is a hurdle of 2D in vitro systems [334, 339]. The culture of pancreatic islets on a microfluidic chip with an optimized flow rate to mimic the interstitial flow conditions has been proven to be effective in maintaining the functionality of the islets and reducing the shear stress‐induced cellular damage [340]. The use of such platforms for pancreatic in vitro direct reprogramming can help in optimizing and refining the reprogramming factors. Matrix stiffness is the other biophysical cue that varies across cell types and plays an important role in regulating cellular behavior. For instance, astrocytes typically require a relatively stiffer substrate compared to neurons, and variations in substrate stiffness have been shown to influence synapse formation in neurons [341, 342]. Thus, microfluidic devices that can mimic cell type‐specific matrix stiffness can further inform the formulation of reprogramming factors. Similarly, electromechanical stimulation of cells recapitulating physiological parameters has been shown to enhance the generation of mature cardiac cells [343], highlighting the importance of such biophysical cues in identifying the right set of reprogramming factors. These studies have highlighted the ability of microfluidic devices to establish biophysical cues mimicking the tissue microenvironment, which can help in the refinement of reprogramming factors and generation of mature cells by activating the right GRN. Furthermore, the microengineered tissues and organoids installed on these devices can be used to develop strategies for precisely targeting specific cell types, examining their effects on non‐target cells, and understanding the impact of biophysical and biochemical cues on direct reprogramming. These platforms can also be used to generate disease models to evaluate reprogramming efficacy after the induction of cellular reparative mechanisms [334, 344].

**Box 3: Organ‐on‐Chips**

Organ‐on‐Chip (OoC) platforms are composed of a miniaturized 3D culture system integrated on a microfluidic device that replicates key aspects of human organ function. The organoid cultures include the relevant cell types of a specific tissue that are spatially arranged to maintain their interactions, thereby mimicking the tissue's histogenesis. Within these 3D organoids, cell‐cell interactions, biochemical, and biophysical cues recreate the native tissue microenvironment, promoting cellular maturation to attain organ‐level functionality. The integration of microfluidic systems, which comprise microchannels to regulate fluid dynamics, allows precise control over the biophysical environment, such as mechanical stress and shear forces. The microfluidic devices can help mimic the tissue‐specific stiffness that promotes cell functionality and enables simulation of physiological fluid dynamics. As a result, OoC systems closely mimic whole‐organ physiology and serve as valuable platforms for studying drug absorption, distribution, metabolism, efficacy, and toxicity. Beyond pharmacological testing, OoCs are increasingly used to model human diseases, investigate molecular mechanisms, and perform high‐throughput drug screening across various cell types. A notable advancement in this field is the development of body‐on‐chip systems, which connect multiple organ models to replicate inter‐organ communication, paracrine signaling, interstitial flow, and systemic drug distribution and metabolism. In the context of direct reprogramming, OoCs offer a sophisticated platform for replicating the complex, dynamic 3D microenvironment found in vivo. These systems allow fine‐tuning of reprogramming protocols by enabling the optimization of reprogramming factors’ dosage, exposure duration, reprogramming factors’ half‐life, and studying the effect of reprogramming factors on non‐target cells within the tissue. Moreover, they facilitate investigation into how local biochemical and biophysical cues may synergize or interfere with reprogramming outcomes.

### 7.4 Mechanistic Understanding of Direct Reprogramming

The advent of single‐cell technologies has significantly advanced our mechanistic understanding of direct reprogramming, facilitated lineage tracing, and elucidated the molecular mechanisms underlying this process. These technologies have enabled the identification of molecular events, reprogramming intermediates, reprogramming trajectories, bifurcation points, and molecular roadblocks, thus paving the way for rapid and efficient reprogramming strategies [260, 345]. Furthermore, the characterization of reprogramming intermediates and trajectories has highlighted the involvement of developmental and regenerative programs in the reprogramming process, giving possible cues to further refine the process [346]. In a recent study, Xing et al. [347] used single‐cell sequencing data to identify the crucial switch from Fosl1‐to Tead4‐centric expression for successful reprogramming, thereby highlighting Fosl1 as a molecular roadblock responsible for the trajectory generation of non‐reprogrammed cells. The identification of such bifurcation points and molecular roadblocks can help in designing protocols with rationalized exposure times or additional reprogramming factors to sustain reprogramming trajectories, thereby generating successfully reprogrammed cells. In this regard, the identification of GRNs governing successful and unsuccessful reprogramming trajectories can be a useful approach. Various machine learning‐based models have been developed to infer GRNs from single‐cell omics data, and their integration with trajectory analysis can help in the identification of GRNs governing successful and unsuccessful reprogramming trajectories [348, 349, 350]. Furthermore, such models can also be used to identify molecular players that govern the induction of specific GRNs, followed by validation using in silico perturbation studies to identify molecular barriers to direct reprogramming [351, 352, 353]. Single‐cell omics data have also identified reprogramming intermediates with youthful signatures and regeneration‐like programs, motivating the concept of in vivo partial reprogramming to reduce physiological age and promote tissue regeneration [283, 354]. Wang et al. [355] recently used the transient expression of the Yamanaka factors to induce partial reprogramming in aged Schwann cells to attenuate senescence‐associated phenotype and functional deficits, thereby promoting regeneration following sciatic nerve injury. Furthermore, a growing understanding of the GRN that can be targeted can lead to the generation of lineage‐restricted progenitor cells, which could serve as a multi‐modal therapeutic intervention. Progenitor cells offer several advantages over terminally differentiated cells in specific contexts. For instance, neural stem cells are preferred over terminally differentiated neurons for treating neurodegenerative diseases because of their ability to modulate immune responses, replace damaged neurons, and rebuild neural networks [356, 357, 358].

### 7.5 Multimodal In Vivo Reprogramming Approaches for Clinical Application

Despite advancements in reprogramming factors and delivery strategies, the translation of in vivo reprogramming to clinical settings remains challenging. This highlights the need for a multimodal approach that integrates various aspects of in vivo reprogramming. Consequently, novel approaches for direct in vivo reprogramming have emerged, demonstrating their potential therapeutic efficacy and clinical applicability. Recent studies have reported promising results in this regard. Kannappan et al. [209] induced adipose browning using a microneedle patch loaded with nanoparticles that encapsulated small molecules (Table 3). Local and sustained delivery of small molecules from this microneedle‐nanoparticle platform restored glucose metabolism in mice fed a high‐fat diet. For cancer immunotherapy, Ascic et al. [359] delivered a set of TFs (Pu.1, Irf8, and Batf3) for in vivo direct reprogramming of fibroblasts and tumor cells to type 1 conventional dendritic cells (cDC1s), which are crucial for T‐cell recruitment and activation, as well as cancer cell elimination. Apart from direct reprogramming approaches to convert differentiated cells from one lineage to another, in vivo manipulation of progenitor cell differentiation or phenotypic and functional modulation of cells has also recently been shown to have potential to promote tissue regeneration. Although this approach does not align with the conventional definition of direct reprogramming, it introduces a promising new avenue for cell‐based therapy. For instance, Chaves‐Perez et al. [360] showed colonic regeneration using α‐ketoglutarate (αKG), a tricarboxylic acid (TCA) cycle intermediate, for lineage specification of intestinal stem cells (ISCs) toward secretory cells. This study sheds light on the possibility of tissue regeneration by targeting and modulating the behavior of tissue‐resident progenitors. With regard to the in vivo phenotypic and functional modulation of cells, Huang et al. [361] mitigated the pro‐inflammatory tumor microenvironment in acute lung injury using an inhalable CRISPR/Cas9 nanotherapeutic platform developed for immune‐metabolic reprogramming of macrophages. Similarly, Kim et al. [285] used poly‐gamma glutamic acid‐based nanoparticles for the specific in vivo delivery of fenofibrate to CD3^+^ T cells for lipid reprogramming, leading to improved T cell proliferation and effector killing effect in a tumor microenvironment.

### 7.6 Toward Clinical Application

The successful clinical translation of in vivo direct reprogramming will not only depend on the optimization of reprogramming strategies, but also on addressing challenges related to scalability, patient variability, production pipeline, and regulatory requirements [362, 363, 364]. Although promising therapeutic outcomes have been demonstrated in preclinical studies, comprehensive long‐term evaluations of reprogrammed cell fate remain limited. Therefore, future studies should emphasize lineage tracing, long‐term assessment of cellular identity and functionality, and safety profiling to facilitate clinical application. In addition, most current studies have been conducted in small animal models such as mice and rats, whose outcomes cannot be directly extrapolated to humans due to differences in delivery efficiency, dosage requirements, and immunogenicity. Consequently, future efforts should incorporate large animal models, including pigs and non‐human primates, to better evaluate delivery strategies and optimize the scaling of both reprogramming factors and delivery vehicles for generating sufficient reprogrammed cells to restore tissue functionality [365, 366]. Furthermore, the development of scalable manufacturing approaches for tissue‐specific reprogramming factors and delivery vehicles will be essential for successful clinical implementation. A standardized and scalable production process must ensure the purity, stability, and batch‐to‐batch consistency in the production of reprogramming factors and the delivery vehicles. For approval for application in a clinical setting, a production pipeline containing quality control measures and in compliance with Good Manufacturing Practice (GMP) will be essential [367].

For first‐in‐human trials, initial studies should primarily focus on evaluating safety, delivery efficiency, route of administration, and real‐time monitoring, as these will serve as critical benchmarks for the clinical translation of in vivo direct reprogramming. Subsequently, studies aimed at optimizing the dosage of both reprogramming factors and delivery vehicles, assessing reprogramming efficiency and therapeutic outcomes, will provide a clearer understanding of the overall efficacy of this approach. Another major challenge in the clinical application of direct reprogramming is patient‐to‐patient variability, as factors including biological age, overall health, immune background, and injury status can substantially influence reprogramming outcomes [22, 368, 369]. Reprogramming cocktails that produce therapeutic benefits in one patient may result in only partial reprogramming, reduced cell survival, or off‐target effects in others. Therefore, establishing well‐defined patient subsets based on these variables will be essential for improving therapeutic consistency and clinical success [370, 371].

Establishing an appropriate regulatory framework will be a critical step for the successful clinical translation of in vivo direct reprogramming. Regulatory bodies emphasize understanding the mechanism of action, evaluating off‐target effects, and conducting long‐term follow‐up studies to monitor the stability and functionality of reprogrammed cells [372, 373]. Other important considerations include the biodistribution and persistence of delivery vehicles and reprogramming factors, cellular potency, and the potential risk of tumorigenicity [373, 374, 375]. Preclinical studies across multiple animal models are an important platform for addressing many of these concerns. Furthermore, because in vivo direct reprogramming typically involves multiple components including delivery vehicles and reprogramming factors, regulatory discussions may need to consider evaluating the integrated platform as a single therapeutic entity, rather than assessing each component independently, since many individual components may be relatively inert on their own [30]. Such an approach could help streamline and accelerate the regulatory approval process.

## 8 Conclusion

In vivo direct reprogramming has emerged as a transformative therapeutic strategy that enables the direct generation of functional and therapeutically active cells within the native tissue microenvironment. This approach not only promotes tissue integration and survival but also addresses the limitations associated with traditional cell transplantation methods. Its clinical potential is increasingly evident in various domains, including neurodegenerative diseases (e.g., Parkinson's disease), metabolic disorders (e.g., type 1 diabetes or obesity), cardiovascular diseases (e.g., myocardial infarction), and pulmonary fibrosis. Notably, in cancer immunotherapy, in situ reprogramming of immune or stromal cells may enhance antitumor responses. However, it is important to note that these advancements have still been confined to preclinical studies, and the application of in vivo direct reprogramming has not reached clinical studies yet. Despite the identification of several reprogramming factors and development of delivery strategies, the clinical translation has been hindered due to reprogramming efficacy, targeting specificity, and safety concerns. The recent advancements, including the integration of disease‐specific reprogramming protocols, advanced delivery vehicles, CRISPR‐based epigenetic modulators, and single‐cell‐resolved analyses, will be pivotal in overcoming the current challenges related to the efficiency, specificity, and safety of in vivo direct reprogramming. These interdisciplinary innovations are poised to expand the therapeutic repertoire, enabling precise organ‐specific regeneration and extending the application of in vivo direct reprogramming to address age‐related degeneration, fibrosis, and oncological conditions. As we continue to refine the reprogramming process and leverage high‐resolution biological data, in vivo direct reprogramming holds great promise for redefining regenerative medicine, moving beyond mere repair to rejuvenation and disease modification, even in contexts previously considered untreatable.

## Author Contributions

**Mauro Calvoli**: writing – original draft, investigation, data curation. **Kyeong Kyu Kim**: conceptualization, supervision, resources, writing – review and editing, funding acquisition. **Rishabh Deo Singh**: conceptualization, visualization, writing – original draft, writing – review and editing, investigation, data curation.

## Funding

This work was supported by grants from the National Research Foundation (RS‐2023‐NR077275 and RS‐2024‐00440289).

## Conflicts of Interest

The authors declare no conflicts of interest.

## Data Availability

The data that support the findings of this study are available from the corresponding author upon reasonable request.
